# Screening of Mediterranean Plant-Derived Extracts for Antioxidant Effect in Cell-Free and Human Cell Line Models

**DOI:** 10.3390/antiox14101217

**Published:** 2025-10-09

**Authors:** Giuseppe Argentino, Edoardo Giuseppe Di Leo, Chiara Stranieri, Stefano Negri, Mauro Commisso, Flavia Guzzo, Anna Maria Fratta Pasini, Annalisa Castagna, Simonetta Friso

**Affiliations:** 1 Department of Medicine, University of Verona, 37134 Verona, Italy; edoardogiuseppe.dileo@univr.it (E.G.D.L.); chiara.stranieri@univr.it (C.S.); annamaria.frattapasini@univr.it (A.M.F.P.); annalisa.castagna@univr.it (A.C.); simonetta.friso@univr.it (S.F.); 2 National Biodiversity Future Center (NBFC), 90133 Palermo, Italy; stefano.negri@univr.it (S.N.); mauro.commisso@univr.it (M.C.); flavia.guzzo@univr.it (F.G.); 3 Department of Biotechnology, University of Verona, 37134 Verona, Italy

**Keywords:** plant extracts, natural antioxidants, Mediterranean flora, oxidative stress, reactive oxygen species, cytotoxicity, redox homeostasis, THP-1 cells, human primary cell models, oxidative stress-related disorders

## Abstract

Oxidative stress plays a critical role in the development of various chronic diseases, leading to major health problems worldwide. There has been increasing interest in using natural antioxidants as complementary agents for maintaining redox homeostasis and assuring a healthy lifestyle. This study aimed to systematically screen the antioxidant potential and cytotoxicity profiles of 19 plant-derived extracts using both a cell-free Fenton reaction-based assay and human monocytic THP-1 cells in vitro. The radical-scavenging capacity varied markedly among the extracts, with *Acalypha virginica* Linnaeus (ACALYPHA), *Acorus calamus* Linnaeus (ACORUS), *Actinidia deliciosa* (A.Chev.) C.F. Liang & A.R. Ferguson (ACTINIDIA), and *Heuchera sanguinea* Pursh (HEUCHERA) demonstrating strong activity in the chemical assay. In the cellular model, 15 extracts significantly reduced intracellular reactive oxygen species (ROS) levels without inducing cytotoxicity at effective concentrations. Notably, *Acalypha virginica* Linnaeus (ACALYPHA), *Actinidia deliciosa* (A.Chev.) C.F. Liang & A.R. Ferguson (ACTINIDIA), *Dianthus superbus* Linnaeus subsp. *superbus* (DIANTHUS), *Succisa pratensis* Moench (SUCCISA), and *Typha laxmannii* Lepech (TYPHA) exhibited consistent antioxidant efficacy across multiple doses. At higher concentrations, all extracts triggered apoptosis and/or necrosis, emphasizing the importance of defining safe ranges. These findings provide a comprehensive comparative analysis of Mediterranean plant-based natural antioxidants obtained by an in vitro approach. The selected plant extracts could be considered as promising candidates for the development of strategies targeting oxidative stress-related disorders. Further investigations considering the specific phytochemical composition of each extract and in vivo validation are needed to confirm their efficacy and safety.

## 1. Introduction

Reactive oxygen species (ROS), including superoxide anion (O_2_^−^), hydrogen peroxide (H_2_O_2_), nitric oxide (NO), and hydroxyl radicals (•OH), are highly reactive molecules involved in numerous physiological and pathological processes [1,2,3,4]. While essential for signaling, immune homeostasis, proliferation, tissue repair, and stress adaptation [5,6,7], excessive ROS disrupt redox balance, impair regenerative processes, and trigger regulated cell death pathways such as apoptosis, necrosis, ferroptosis, and proptosis [8,9,10,11,12,13]. Thus, maintaining ROS at optimal levels is crucial for cellular integrity.

Organisms regulate ROS through complex antioxidant networks [14]. Enzymatic antioxidants—superoxide dismutase (SOD), catalase (CAT), and glutathione peroxidase (GPx)—act synergistically with non-enzymatic scavengers such as vitamins C and E, β-carotene, coenzyme Q10, selenium, and zinc to preserve redox balance without eliminating ROS completely [15,16]. This modulation supports vital cellular functions while preventing oxidative damage.

Chronic oxidative stress contributes to cardiovascular diseases, diabetes, neurodegeneration, and cancer [17,18]. ROS-induced damage affects proteins, lipids, and nucleic acids, promoting inflammation, mitochondrial dysfunction, and cell death [17,18,19,20,21,22]. Neurons are especially vulnerable due to their high oxygen consumption and limited regeneration capacity [17,18,19,20,21]. Accordingly, antioxidant strategies targeting ROS offer promising avenues for prevention and therapeutic intervention across a range of chronic diseases.

Plants represent a prolific and sustainable source of bioactive compounds with considerable pharmaceutical potential. Land plants are capable of synthesizing an extensive array of metabolites, commonly known as phytochemicals, with diverse and complex chemical structures and functions. The ecological interactions in which plants are embedded often drive the biosynthesis of these compounds, which can subsequently be extracted, isolated, and purified using both conventional and innovative methodologies [23]. While the selection of plant materials for research and development is frequently guided by availability, this empirical strategy proves especially fruitful in regions characterized by high biodiversity and endemism, where chemical diversity often reflects the ecological richness of the source organisms. This approach is grounded in the theory that secondary metabolites that evolved to serve specific ecological purposes, such as signaling and protection, may interact beneficially with human biological systems, owing to the evolutionary conservation of core biochemical architectures [24]. Phytochemicals are generally classified into two broad categories: primary constituents and secondary (specialized) metabolites. Primary plant constituents such as sugars, amino acids, proteins, chlorophyll, and nucleic acids are essential for metabolism and structural integrity. In contrast, specialized metabolites (including, for instance, alkaloids, flavonoids, terpenes, and phenolic compounds) are not directly involved in primary metabolism but are crucial for plant defense and environmental adaptation (e.g., against herbivores, pathogens, ultraviolet radiation, and other stressors). Many of these compounds display notable antioxidant properties. While not essential for human physiology, phytochemicals have shown diverse health-promoting effects, including antioxidant, anti-inflammatory, antimicrobial, anticancer, and neuroprotective activities [18,25]. Their ability to scavenge free radicals and modulate redox-sensitive pathways highlights their potential in preventing and managing oxidative stress-related diseases such as cardiovascular and neurodegenerative disorders, and cancer [26,27]. Consequently, the growing scientific interest in phytochemicals spans across disciplines, from pharmacology to nutrition science, highlighting their potential not only as therapeutic agents but also as functional ingredients in preventive healthcare and as basic diet constituents for a healthy lifestyle [28].

Based on the growing interest in the antioxidant properties of phytochemicals, the aim of this study is to identify species with notable antioxidant activity among plant species collected within the scope of the “National Biodiversity Future Center (NBFC)” project (https://www.nbfc.it/en). The species explored for this investigation represent a broad and diverse selection of plants, encompassing different ecological origins, including wild, medicinal, aromatic, industrial, and urban biomass sources, as well as a wide biochemical potential. These species were chosen with a view to maximize the coverage of different plant families, capture distinct metabolomic profiles, and prioritize extracts enriched in rare or underexplored compounds. This approach ensures that the species studied reflect the diversity of Italian flora and possess the potential to identify novel plant-derived compounds with antioxidant activity. Through this targeted analysis, the project also aims to provide a comprehensive resource on bioactive phytochemicals and explore their potential applications in promoting human health, preventing oxidative stress-related disorders, and supporting the sustainable and biodiversity-conscious utilization of Mediterranean plant resources.

## 2. Materials and Methods

### 2.1. Selection of Plant Species, Extract Preparation, and Phytochemical Characterization

The selection of plant species was based on the official checklist of the Italian flora, available through the Portal of Italian Flora (available at: https://dryades.units.it/floritaly/index.php, accessed on 1 November 2022), which includes both native and non-native species, comprising naturalized and non-naturalized taxa. For this project, we selected 19 species starting from an initial selection of 720, which was then further refined to 200, based on NBFC criteria (available at: https://www.nbfc.it/en, accessed on 1 November 2022). The species included in this study were *Acalypha virginica* Linnaeus (ACALYPHA; Euphorbiaceae), *Acorus calamus* Linnaeus (ACORUS; Acoraceae), *Actinidia deliciosa* (A.Chev.) C.F. Liang & A.R. Ferguson (ACTINIDIA; Actinidiaceae), *Adenophora liliifolia* (L.) Gaudin (ADENOPHORA; Campanulaceae), *Akebia quinata* (Thunberg ex Houttuyn) Decaisne (AKEBIA; Lardizabalaceae), *Allium lusitanicum* (L.) Mertens (ALLIUM; Amaryllidaceae), *Althaea officinalis* Linnaeus (ALTHAEA; Malvaceae), *Aquilegia atrata* Wilhelm Daniel Joseph Koch (AQUILEGIA; Ranunculaceae), *Ceratophyllum demersum* Linnaeus (CERATOPHYLLUM; Ceratophyllaceae), *Cistus monspeliensis* Linnaeus (CISTUS; Cistaceae), *Dianthus superbus* Linnaeus subsp. *superbus* (DIANTHUS; Caryophyllaceae), *Empetrum hermaphroditum* Hagerup (EMPETRUM; Ericaceae), *Eryngium maritimum* Linnaeus (ERYNGIUM; Apiaceae), *Heuchera sanguinea* Pursh (HEUCHERA; Saxifragaceae), *Iris pseudacorus* Linnaeus (IRIS; Iridaceae), *Petasites paradoxus* (Retzius) Baumgarten (PETASITES; Asteraceae), *Salvia pratensis* Linnaeus (SALVIA; Lamiaceae), *Succisa pratensis* Moench (SUCCISA; Dipsacaceae), and *Typha laxmannii* Lepech (TYPHA; Typhaceae). Plant material (leaves and herbaceous stems) from each species was collected during the vegetative growth phase. Extracts were obtained using previously established protocols [29,30]. In brief, 1 g of frozen powdered plant material was extracted with 10 volumes (*w*/*v*) of LC-MS grade methanol, vortexed for 30 s, and sonicated on ice in a 40 kHz ultrasonic bath (Soltec, Milano, Italy) for 10 min. Following centrifugation, the extract was diluted with LC-MS-grade water and injected into a UPLC-ESI-HRMS system for phytochemical profiling with the instruments and methods previously described [31,32]. Briefly, extract separation and phytochemical detection were performed via RP C18 chromatography followed by MS analysis performed in FAST-DDA mode in negative and positive ionization [31,32]. Metabolite annotation was supported by the comparison of three orthogonal parameters (*m*/*z* and isotopic distribution, retention time, and fragmentation pattern) with an in-house library of authentic standard compounds and with the information available in public MS databases or in the literature.

Other aliquots of the plant extracts obtained were dried with a speed-vac system (Heto-Holten; Frederiksborg, Denmark) and re-solubilized in an equal volume of ethanol, compatible with the following cell assays.

### 2.2. Fenton Reaction

The Fenton reaction was performed as previously described by Wardman and Candeias [33] with slight modifications to assess the ability of plant extracts to modulate or trigger intrinsic free radical production without the mediation of the cellular “system”. In brief, ammonium iron (II) sulfate (Merck KGaA, Darmstadt, Germany) was used as a reference standard for the reaction titration at a concentration of 3.55 µM. Prior to use, ammonium iron (II) sulfate was oxidized in air and stored in small aliquots at −80 °C. Different preparations containing ammonium iron (II) sulfate were combined with increasing concentrations of plant extract (1–100 µg/mL). Following the addition of ascorbic acid (20 µM) (Merck KGaA) and the fluorescent dye dihydrorhodamine 123 (50 µM) (Merck KGaA), all samples were monitored every 3 min for 60 min. Each sample was analyzed in triplicate using a fluorescence plate reader (Fluoroskan Ascent, Thermo Electron Corporation, Vantaa, Finland; excitation at 485 nm, emission at 520 nm).

### 2.3. Cell Cultures

This study was conducted using three distinct human cell lines: human monocytic leukemia cells (THP-1; AddexBio, San Diego, CA, USA), human umbilical vein endothelial cells (HUVECs; ScienCell Research Laboratories, Carlsbad, CA, USA), and human primary small intestinal epithelial cells (HIECs; Cell Biologics, Chicago, IL, USA). THP-1 cells were cultured in RPMI 1640 medium (AddexBio) supplemented with 10% fetal bovine serum (FBS; Sigma-Aldrich, St. Louis, MO, USA), 1% penicillin–streptomycin solution, 0.1% amphotericin B, 0.1% tylosin, and 0.5% heparin, all obtained from Thermo Fisher Scientific (Waltham, MA, USA). HUVECs were maintained in endothelial cell medium (ScienCell Research Laboratories) supplemented with 5% FBS, an endothelial cell growth supplement (ScienCell Research Laboratories), and 1% penicillin–streptomycin. HIECs were cultured in human epithelial cell medium (Cell Biologics) with the addition of 5% FBS, a proprietary epithelial cell medium supplement (Cell Biologics), and 1% penicillin–streptomycin. All cell lines were incubated at 37 °C in a humidified atmosphere containing 5% CO_2_ and subcultured using Accutase (Millipore, Billerica, MA, USA). For non-immortalized cell lines, including HUVECs and HIECs, experimental procedures were conducted using cells between passages 4 and 10 to maintain consistency and ensure cell viability.

### 2.4. Cell Viability Assay

To identify a plant extract concentration range that did not elicit apoptotic or necrotic effects in cultured cells, the PE Annexin V Apoptosis Detection Kit I (AnnV; BD Pharmingen/BD Biosciences, San Jose, CA, USA) was employed following the manufacturer’s protocol. Cells were seeded in 24-well plates at a density of 1 × 10^6^ cells/mL and exposed to increasing concentrations of plant extract for 16–18 h. Following incubation, cell viability and the extent of apoptosis were assessed via flow cytometry using a FACSCelesta (BD Biosciences, Franklin Lakes, NJ, USA).

### 2.5. Intracellular ROS Measurement

The intracellular accumulation of ROS was quantified using the CellROX™ Deep Red Flow Cytometry Assay Kit (Molecular Probes/Thermo Fisher Scientific, Waltham, MA, USA), following the protocol provided by the manufacturer. Cells were seeded in 24-well plates at a density of 5 × 10^5^ cells/mL and treated overnight with the selected plant extracts. Post-incubation, cells were rinsed with phosphate-buffered saline (PBS) and exposed to 200 µM tert-butyl hydroperoxide (TBHP; Sigma-Aldrich) for 45 min to serve as a positive control for oxidative stress. Subsequently, cells were stained with 500 nM CellROX™ Deep Red reagent to detect ROS production and analyzed by flow cytometry. All nineteen plant extracts were initially screened in THP-1 cells. Based on their ROS-modulatory effects, the three most active extracts were then further tested in HUVEC and HIEC cell lines.

### 2.6. Statistical Analysis

The data were analyzed using GraphPad Prism software (Version 10, GraphPad Software Inc., La Jolla, CA, USA) for statistical evaluation. The results are presented as the mean ± standard deviation (SD) of oxidative stress percentages from at least three independent experiments, each including three biological replicates and three technical replicates, with the positive control set at 100% oxidative stress. Statistical comparisons were performed using one-way analysis of variance (ANOVA), with significance determined at *p* < 0.05.

## 3. Results

### 3.1. Phytochemical Characterization

An untargeted metabolomic analysis was performed in order to elucidate the major phytochemicals characterizing the extracts of the plant species under investigation. As expected, the species exhibited very diversified phytocomplexes, with distinct classes of metabolites accumulating in each extract (Table 1 and Supplementary Files S1 and S2).

### 3.2. Cell-Free Radical-Scavenging Activity

**Figure 1:**
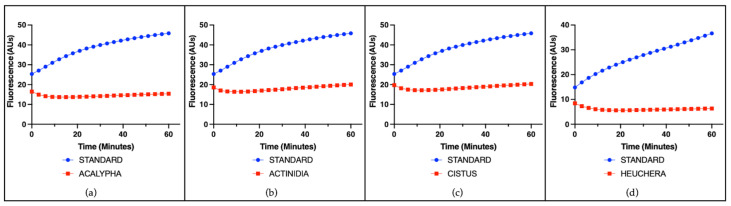
Radical-scavenging activity of plant extracts. Representative fluorescence (arbitrary units) over time induced by the standard iron (II) and by individual plant extracts (25 μg/mL) in the ascorbate-driven Fenton reaction after 60 min. Strong radical-scavenging (red line) activity was observed for (**a**) ACALYPHA, (**b**) ACTINIDIA, (**c**) CISTUS, and (**d**) HEUCHERA.

**Figure 2:**
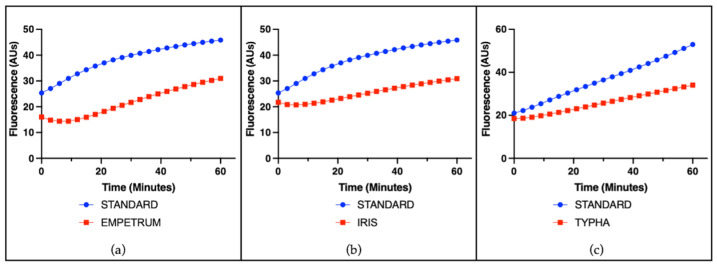
Radical-scavenging activity of plant extracts. Representative fluorescence (arbitrary units) over time induced by the standard iron (II) and by individual plant extracts (25 μg/mL) in the ascorbate-driven Fenton reaction after 60 min. Moderate activity (red line) was observed for (**a**) EMPETRUM, (**b**) IRIS, and (**c**) TYPHA.

**Figure 3:**
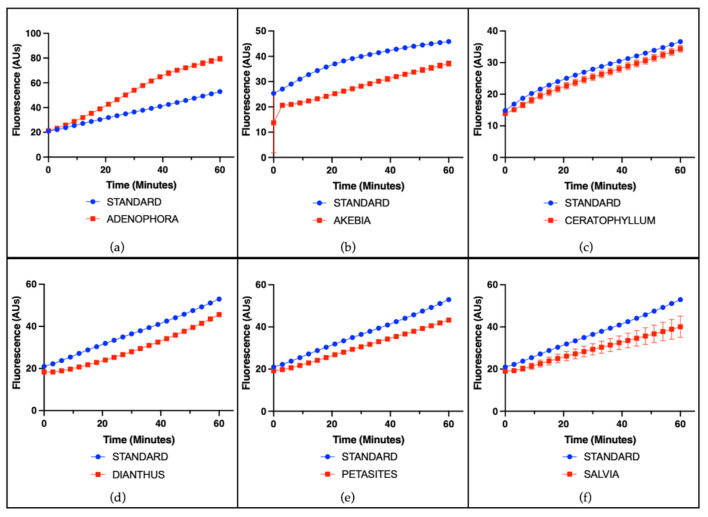
Radical-scavenging activity of plant extracts. Representative fluorescence (arbitrary units) over time induced by the standard iron (II) and by individual plant extracts (25 μg/mL) on the ascorbate-driven Fenton reaction after 60 min. Low activity (red line) was observed for (**a**) ADENOPHORA, (**b**) AKEBIA, (**c**) CERATOPHYLLUM, (**d**) DIANTHUS, (**e**) PETASITES, and (**f**) SALVIA.

**Figure 4:**
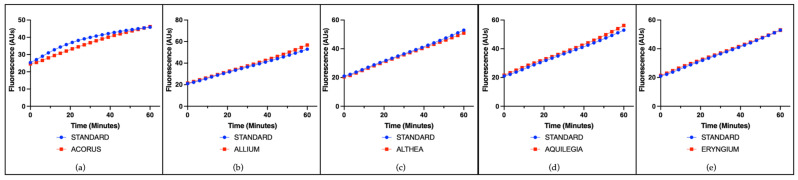
Radical-scavenging activity of plant extracts. Representative fluorescence (arbitrary units) over time induced by the standard iron (II) and by individual plant extracts (25 μg/mL) on the ascorbate-driven Fenton reaction after 60 min. No relevant activity was recorded for (**a**) ACORUS, (**b**) ALLIUM, (**c**) ALTHEA, (**d**) AQUILEGIA, or (**e**) ERYNGIUM.

The radical-scavenging capacity of single plant extracts was evaluated using a cell-free assay based on the ascorbate-driven Fenton reaction, which enables the assessment of intrinsic antioxidant activity independently of cellular components. A representative example of the results for all species is shown using a concentration of 25 µg/mL. Among the tested extracts, ACALYPHA, ACTINIDIA, CISTUS, and HEUCHERA exhibited high radical-scavenging activity (Figure 1), reflecting a strong intrinsic antioxidant potential. EMPETRUM, IRIS, and TYPHA showed moderate scavenging capacity (Figure 2), indicating a partial efficacy in neutralizing free radicals under the applied conditions. In contrast, ADENOPHORA, AKEBIA, CERATOPHYLLUM, DIANTHUS, PETASITES, and SALVIA demonstrated only low activity levels (Figure 3), suggesting a limited antioxidant profile in this experimental setting. The remaining extracts did not display any significant radical-scavenging effect (Figure 4), suggesting the absence of active constituents capable of counteracting ROS generation in this non-cellular system.

### 3.3. Effect of Plant Extract on Cell Viability

The AnnV assay was used to assess cell viability and to determine the appropriate concentration range for subsequent antioxidant activity analyses. Since the data indicated that all extracts, ranging from 0.5 to 25 µg/mL medium, did not induce apoptosis and/or necrosis in THP-1 cells, the subsequent experiments were performed using this concentration range. However, cytotoxicity was observed at a concentration of 250 µg/mL (Supplementary Figure S1a). Non-immortalized cells exhibited cytotoxic responses at the following concentrations: 25 µg/mL in HUVECs (Supplementary Figure S1b) and 150 µg/mL in HIECs (Supplementary Figure S1c).

### 3.4. Evaluation of Antioxidant Activity

**Figure 5:**
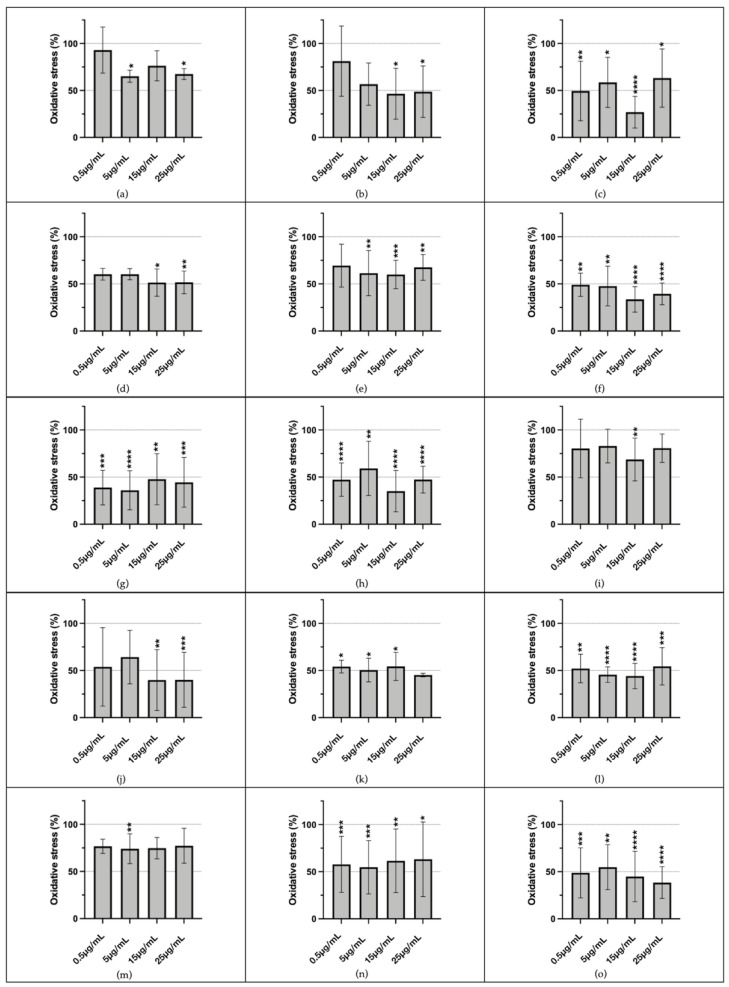
Antioxidant potential of plant extracts in THP-1 cells. Significant antioxidant effects, indicated by a reduction in intracellular ROS levels compared to the positive control, were observed in (**a**) ACALYPHA at 5 and 25 µg/mL; (**b**) ACTINIDIA at 15 and 25 µg/mL; (**c**) ADENOPHORA at 0.5, 5, 15, and 25 µg/mL; (**d**) AKEBIA at 15 and 25 µg/mL; (**e**) ALLIUM at 5, 15, and 25 µg/mL; (**f**) ALTHEA at 0.5, 5, 15, and 25 µg/mL; (**g**) AQUILEGIA at 0.5, 5, 15, and 25 µg/mL; (**h**) DIANTHUS at 0.5, 5, 15, and 25 µg/mL; (**i**) EMPETRUM at 15 µg/mL; (**j**) ERYNGIUM at 15 and 25 µg/mL; (**k**) IRIS at 0.5, 5, and 15 µg/mL; (**l**) PETASITES at 0.5, 5, 15, and 25 µg/mL; (**m**) SALVIA at 5 µg/mL; (**n**) SUCCISA at 0.5, 5, 15, and 25 µg/mL; and (**o**) TYPHA at 0.5, 5, 15, and 25 µg/mL (* *p* < 0.05, ** *p* < 0.01, *** *p* < 0.001, **** *p* < 0.0001).

The assessment of the antioxidant potential of the tested plant-derived extracts in THP-1 cells revealed promising effects. Among the 19 extracts analyzed, only four (ACORUS, CERATOPHYLLUM, CISTUS, and HEUCHERA) did not induce a statistically significant reduction in intracellular ROS production. The remaining extracts exhibited varying degrees of antioxidant activity at different concentrations (Figure 5), as detailed in Supplementary Table S1.

The antioxidant effect in primary non-immortalized cells (HUVECs and HIECs), given the results in THP-1 cells, was limited to three extracts (ALTHEA, AQUILEGIA, and ERYNGIUM), and only ERYNGIUM at 100 µg/mL induced a statistically significant decrease in ROS production (5.94% ± 3.51, *p* = 0.005) (Supplementary Figure S2).

## 4. Discussion

In this study, we evaluated the antioxidant potential of a series of plant extracts using two complementary approaches: a cell-free system based on the Fenton reaction and a cellular model employing human monocytic THP-1 cells, human umbilical vein endothelial cells, and human primary small intestinal epithelial cells. The results highlighted the ability to neutralize ROS, to a significant extent, of several extracts, with variability depending on the assay system and the tested concentration.

The untargeted metabolomic analysis revealed a wide spectrum of secondary metabolites with well-documented biological activities, which may plausibly explain the bioactivities observed in the extracts. For instance, flavonoids (flavonols, flavones, C-glycosylated flavonoids), detected in ACORUS, AQUILEGIA, SUCCISA, and EMPETRUM, are recognized for their strong antioxidant and anti-inflammatory potential, often acting through the modulation of signaling pathways such as NF-κB and MAPK, inhibition of pro-inflammatory enzymes (COX-2, iNOS), and suppression of cytokine release (IL-6, TNF-α) [34]. Proanthocyanidins, highly abundant in ACTINIDIA and TYPHA, are well known for their antioxidant, antimicrobial, and cardioprotective effects, as well as their ability to prevent lipid peroxidation and support cellular integrity [33]. Pentacyclic triterpenoids (oleanane, ursane, taraxastane types), detected in AKEBIA, ACTINIDIA, ERYNGIUM, and DIANTHUS, are reported to exert anti-inflammatory and cytoprotective effects, partly by downregulating pro-inflammatory mediators (NO, TNF-α, IL-6, IL-1β) and inhibiting NF-κB nuclear translocation [35]. Similarly, steroidal saponins, as observed in ALLIUM, are widely associated with immunomodulatory, antifungal, and cholesterol-lowering properties, thereby possibly contributing synergistically to the bioactivity profiles [36]. Phenolic acids and hydrolysable tannins (gallic acid derivatives, ellagitannins), characterizing ACALYPHA, CISTUS, and HEUCHERA, are also well established for their potent radical-scavenging capacity and antimicrobial potential, which may play a central role in the extracts’ antioxidant activities. In Salvia, the detection of rosmarinic acid and related phenylethanoids is particularly relevant, as these compounds are strongly linked to anti-inflammatory and neuroprotective mechanisms [37]. It is also noteworthy that certain species, such as ADENOPHORA and PETASITES, were found to contain pyrrolidine and pyrrolizidine alkaloids. While such alkaloids may possess pharmacological activity, the toxicological implications, particularly hepatotoxicity and genotoxicity, are also well documented, suggesting that their role in the overall bioactivity of these extracts requires careful evaluation [38].

In the cell-free system, extracts such as ACALYPHA, ACTINIDIA, CISTUS, and HEUCHERA exhibited strong radical-scavenging activity, suggesting a substantial intrinsic antioxidant capacity. Previous studies have shown that polyphenols and flavonoids are major contributors to the scavenging activity observed in many plant extracts [39]. These findings are in line with reports that flavonoids such as luteolin and anthocyanins can reduce oxidative stress and inflammation in diverse cell types, including pulmonary cells, red blood cells, and endothelial cells, suggesting a broad-spectrum protective role against ROS-mediated tissue damage [40,41,42]. Other extracts, including EMPETRUM, IRIS, and TYPHA, demonstrated moderate activity, whereas several others did not significantly inhibit radical generation.

Subsequently, we tested these observations in a cellular context, and we were able to confirm that multiple extracts retained antioxidant efficacy under more biologically complex conditions. In THP-1 cells, extracts such as ACALYPHA, ACTINIDIA, DIANTHUS, SUCCISA, and TYPHA significantly reduced intracellular ROS levels at non-cytotoxic concentrations. This is consistent with previous findings suggesting that specific plant extracts can modulate intracellular redox homeostasis [43]. Similar protective effects have been documented in studies where flavonoids mitigated oxidative stress in COPD (chronic obstructive pulmonary disease) models, RBCs exposed to oxidative agents, and diabetic endothelial cells, highlighting the translational potential of these natural antioxidants across multiple disease contexts [40,41,42].

These results are particularly relevant considering accumulating evidence that natural antioxidants derived from medicinal plants may offer effective, low-toxicity strategies for addressing oxidative stress-mediated pathologies [44]. The ability of plant extracts to act at the cellular level supports their translational potential in future therapeutic applications. For instance, luteolin in COPD models was shown to modulate TRPV1/SIRT6 and CYP2A13/NRF2 signaling pathways, reducing ROS production and inflammation [40]. Anthocyanin-enriched fractions protected RBCs from oxidative stress by preserving band 3 function and reducing lipid peroxidation [42]. Moreover, flavonoids have been observed to counteract endothelial dysfunction in diabetes through ROS scavenging and modulation of nitric oxide availability [41].

Cytotoxicity assessment by an AnnV assay revealed that, at high concentrations (250 µg/mL), all extracts induced apoptosis and/or necrosis in THP-1 cells. Notably, primary non-immortalized cells, such as HUVECs and HIECs, exhibited cytotoxic responses at even lower concentrations (25 µg/mL and 150 µg/mL, respectively), underscoring the importance of carefully defining the concentration range in the therapeutic window [45]. Despite some cytotoxicity, antioxidant effects were detectable at relatively low doses. Comparing these effective in vitro concentrations with the known bioactivity and realistic dietary intake of the main phytochemicals in these extracts could provide further insight into their translational potential. However, defining a precise therapeutic window is not possible from this initial screening, which was intended to identify promising candidates for further investigations.

Biologically, the capacity to reduce intracellular ROS levels in monocytes like THP-1 cells is of particular interest, as oxidative stress is a known promoter of immune dysfunction and chronic inflammatory diseases [46], especially atherosclerosis. THP-1 cells are in fact a model of atherosclerosis because they mimic the behavior of foam cells, the leading type of cells in the progression of atherosclerotic lesions.

Indeed, the link between oxidative imbalance and chronic inflammation, neurodegeneration, and cardiovascular damage is well established, making it essential to discover compounds capable of modulating redox homeostasis effectively and safely. Our findings contribute to this growing area of research and support the exploration of plant-derived molecules as adjuvants or leads in pharmaceutical development. These observations align with evidence from animal studies where plant-derived antioxidants such as citric acid reduced LPS-induced oxidative stress and tissue damage in the brain and liver [47].

Identifying effective and safe plant extracts could open new therapeutic avenues for the management of redox-related disorders such as atherosclerosis, diabetes, and neurodegenerative diseases. Moreover, in vivo and in vitro studies have demonstrated that flavonoids and anthocyanins not only reduce ROS but also modulate key signaling pathways (e.g., NRF2, SIRT6, TRPV1) involved in oxidative stress responses, inflammation, and apoptosis, reinforcing their potential as multi-target agents [40,41,42].

Furthermore, in vitro studies on neuronal models have shown that several plant extracts can attenuate oxidative stress-induced apoptosis, pointing to their neuroprotective effects and further underscoring the clinical relevance of identifying such compounds [48]. These insights are consistent with the ROS-reducing capabilities observed in this study, which may be extended to various disease models in future investigations.

However, this study has some limitations. First, antioxidant activity was assessed under acute conditions and in monoculture systems; thus, long-term effects and interactions within complex microenvironments remain to be investigated [49]. Secondly, the challenge in dealing with such different and rich phytocomplexes hinders the attribution of the observed effects to specific phytochemicals.

To address these aspects, fractionation of the plant extracts to test specific groups of phytochemicals from the original phytocomplex might represent a useful approach for identifying the molecule/molecules responsible for the observed bioactivities [50]. Additionally, it will be crucial to explore molecular mechanisms, such as the activation of the Nrf2/ARE pathway, a key player in cellular antioxidant responses [51]. This approach mirrors studies where modulation of NRF2 and SIRT6 signaling by flavonoids and anthocyanins was associated with reduced oxidative stress and cellular protection [40,41,42].

Finally, future studies using animal models of chronic oxidative stress and inflammation will be essential to deepen our knowledge on the potential of these extracts in specific, physiologically relevant settings.

## 5. Conclusions

This study systematically investigated the antioxidant potential of various plant extracts using both a cell-free Fenton reaction-based assay and a human monocytic THP-1 cell model. The findings demonstrate that several extracts, particularly ACALYPHA, ACTINIDIA, DIANTHUS, SUCCISA, and TYPHA, all characterized by very different phytocomplexes, possess significant radical-scavenging and intracellular ROS-reducing activities, without causing cytotoxicity at effective concentrations.

By contributing to broadening the knowledge in the growing field related to the function of natural antioxidants, the findings of this study highlight the potential of novel Mediterranean plant extracts as potential candidates for the development of health-promoting strategies, eventually useful against oxidative stress-related diseases.

Nevertheless, further research is indeed warranted to elucidate their phytochemical composition, molecular mechanisms of action, safety profiles, and bioavailability in vivo.

Importantly, the dual approach adopted in this study, combining a biochemical assay and a cellular model, allowed for a more comprehensive evaluation of antioxidant efficacy under both simplified and biologically relevant conditions. Moreover, the identification of active extracts among species native or naturalized within the Italian flora emphasizes the untapped pharmacological potential of regional biodiversity. By focusing on plant-derived antioxidants with both efficacy and acceptable cytotoxicity profiles, this research contributes valuable knowledge to the field of redox biology and supports the role of phytochemicals in future human health-preserving policies.

## 6. Patents

A patent application related to the antioxidant plant extracts screened in this study has been filed and is currently under review.

## Figures and Tables

**Table 1:** Plant species and characterizing metabolite classes of the phytochemicals detected in the methanolic extracts following untargeted metabolomic analysis.

Species	Characterizing Metabolites
ACALYPHA	gallic acid derivatives, hydrolysable tannins
ACORUS	C-glycosylated flavonoids
ACTINIDIA	proanthocyanidins, flavonols, ursane/taraxastane triterpenoids
ADENOPHORA	flavonoids, pyrrolidine alkaloids
AKEBIA	flavonols, oleanane triterpenoids
ALLIUM	steroidal saponins
ALTHAEA	norisoprenoids sulfoglycosides, hypoleatin derivatives
AQUILEGIA	swertisin derivatives, cycloartane triterpenoids
CERATOPHYLLUM	hydroxycinnamates, flavonoids
CISTUS	ellagitannins, flavonols/methoxylated flavonols, labdane diterpenoids
DIANTHUS	flavonoids, oleanane triterpenoids
EMPETRUM	quercetin derivatives, flavanones, dihydrochalcones
ERYNGIUM	flavonols, triterpenoids
HEUCHERA	gallotannins
IRIS	xanthones, flavones
PETASITES	hydroxycinnamate esters, otonecine-type pyrrolizidine alkaloids
SALVIA	rosmarinic acid and related phenylethanoid/phenylpropanoid esters
SUCCISA	iridoids, flavones
TYPHA	proanthocyanidins, flavonols

## Data Availability

The data presented in this study are available on request from the corresponding author. The data are not publicly available due to ongoing research activities and a pending patent application.

## Supplementary Materials

The following supporting information can be downloaded at: https://www.mdpi.com/article/10.3390/antiox14101217/s1, Table S1: Antioxidant activity of plant extract in THP-1 cells at concentration showing statistically significant effects; Figure S1: Example of the effect of a plant extract on THP-1 cells (a), HUVECs (b), and HIECs (c); Figure S2: Antioxidant potential of ERYNGIUM extracts in HIECs; Supplementary File S1: UPLC-ESI-HRMS chromatograms recorded in negative ionization mode (ESI-) of the plant extracts; Supplementary File S2: LC-MS features of the phytochemicals identified in the plant extracts.

## Abbreviations

The following abbreviations are used in this manuscript:

ROS	reactive oxygen species
SOD	superoxide dismutase
CAT	catalase
GPx	glutathione peroxidase
ACALYPHA	*Acalypha virginica* Linnaeus
ACORUS	*Acorus calamus* Linnaeus
ACTINIDIA	*Actinidia deliciosa* (A.Chev.) C.F. Liang & A.R. Ferguson
ADENOPHORA	*Adenophora liliifolia* (L.) Gaudin
AKEBIA	*Akebia quinata* (Thunberg ex Houttuyn) Decaisne
ALLIUM	*Allium lusitanicum* (L.) Mertens
ALTHAEA	*Althaea officinalis* Linnaeus
AQUILEGIA	*Aquilegia atrata* Wilhelm Daniel Joseph Koch
CERATOPHYLLUM	*Ceratophyllum demersum* Linnaeus
CISTUS	*Cistus monspeliensis* Linnaeus
DIANTHUS	*Dianthus superbus* Linnaeus subsp. *superbus*
EMPETRUM	*Empetrum hermaphroditum* Hagerup
ERYNGIUM	*Eryngium maritimum* Linnaeus
HEUCHERA	*Heuchera sanguinea* Pursh
IRIS	*Iris pseudacorus* Linnaeus (synonim *Limniris pseudacorus*)
PETASITES	*Petasites paradoxus* (Retzius) Baumgarten
SALVIA	*Salvia pratensis* Linnaeus
SUCCISA	*Succisa pratensis* Moench
TYPHA	*Typha laxmannii* Lepech
THP-1	human monocytic leukemia cells
HUVECs	human umbilical vein endothelial cells
HIECs	human primary small intestinal epithelial cells
FBS	fetal bovine serum
AnnV	Annexin V
TBHP	tert-butyl hydroperoxide
UPLC	ultra-pressure liquid chromatography
ESI	electro-spray ionization
HRMS	High-resolution mass spectrometry
COPD	chronic obstructive pulmonary disease
