# Supplementary material for: Screening of Mediterranean Plant-Derived Extracts for Antioxidant Effect in Cell-Free and Human Cell Line Models
Source: Antioxidants (Basel). 2025 Oct 9;14(10):1217. doi: 10.3390/antiox14101217 (PMC12561960; doi:10.3390/antiox14101217)
Supplement: Supplementary file 1 [file antioxidants-14-01217-s001.zip › Supplementary File S1.pptx]

## Slide 1
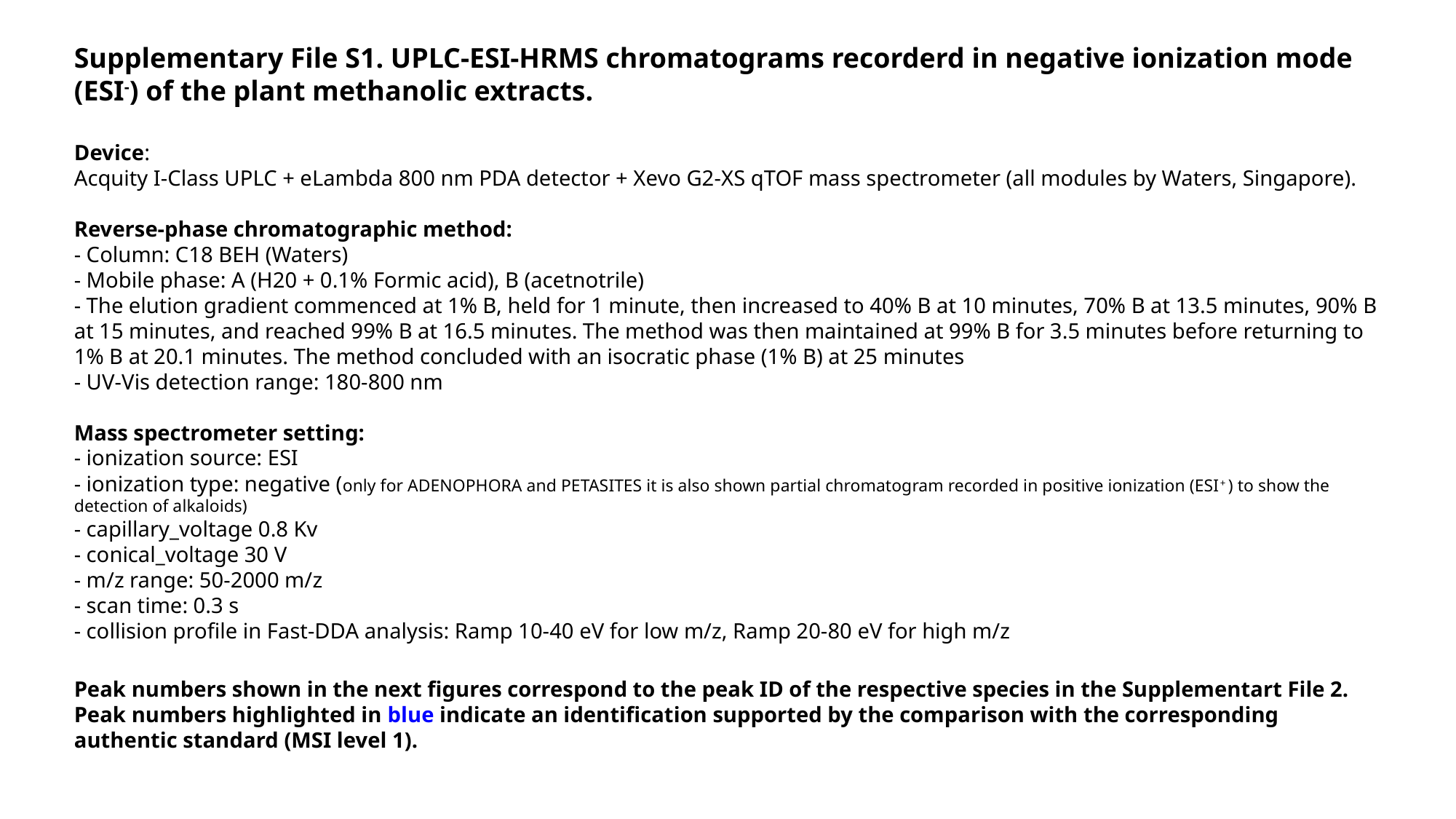

Supplementary File S1. UPLC-ESI-HRMS chromatograms recorderd in negative ionization mode (ESI-) of the plant methanolic extracts.
Device:
Acquity I-Class UPLC + eLambda 800 nm PDA detector + Xevo G2-XS qTOF mass spectrometer (all modules by Waters, Singapore).
Reverse-phase chromatographic method:
- Column: C18 BEH (Waters)
- Mobile phase: A (H20 + 0.1% Formic acid), B (acetnotrile)
- The elution gradient commenced at 1% B, held for 1 minute, then increased to 40% B at 10 minutes, 70% B at 13.5 minutes, 90% B at 15 minutes, and reached 99% B at 16.5 minutes. The method was then maintained at 99% B for 3.5 minutes before returning to 1% B at 20.1 minutes. The method concluded with an isocratic phase (1% B) at 25 minutes
- UV-Vis detection range: 180-800 nm
Mass spectrometer setting:
- ionization source: ESI
- ionization type: negative (only for ADENOPHORA and PETASITES it is also shown partial chromatogram recorded in positive ionization (ESI+ ) to show the detection of alkaloids)
- capillary_voltage 0.8 Kv
- conical_voltage 30 V
- m/z range: 50-2000 m/z
- scan time: 0.3 s
- collision profile in Fast-DDA analysis: Ramp 10-40 eV for low m/z, Ramp 20-80 eV for high m/z
Peak numbers shown in the next figures correspond to the peak ID of the respective species in the Supplementart File 2. Peak numbers highlighted in blue indicate an identification supported by the comparison with the corresponding authentic standard (MSI level 1).

## Slide 2
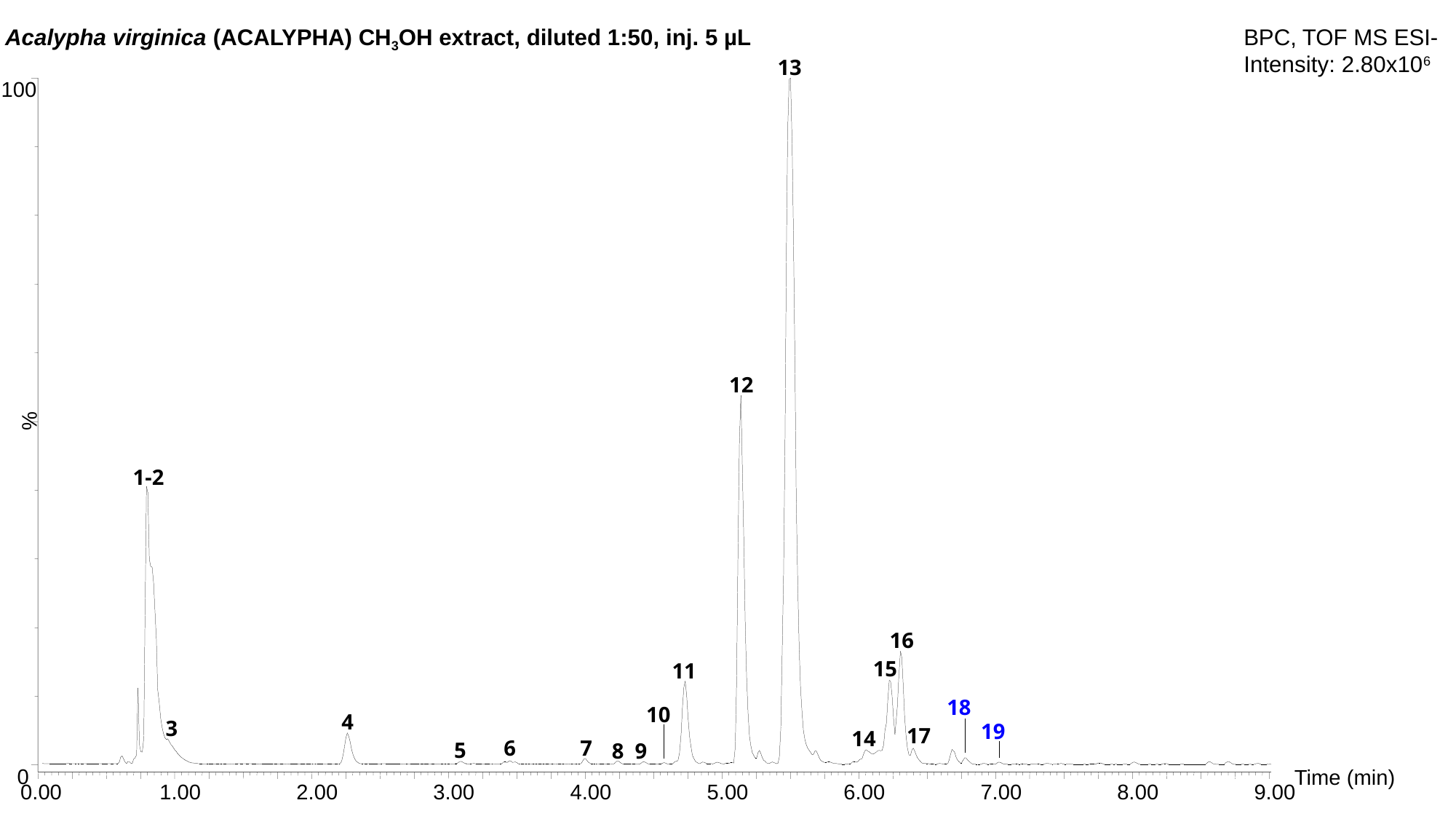

Acalypha virginica (ACALYPHA) CH3OH extract, diluted 1:50, inj. 5 µL
BPC, TOF MS ESI-
Intensity: 2.80x10^6^
13
100
12
%
1-2
16
15
11
18
10
4
3
19
17
14
7
6
5
9
8
0
Time (min)
0.00
1.00
2.00
3.00
4.00
5.00
6.00
7.00
8.00
9.00

## Slide 3
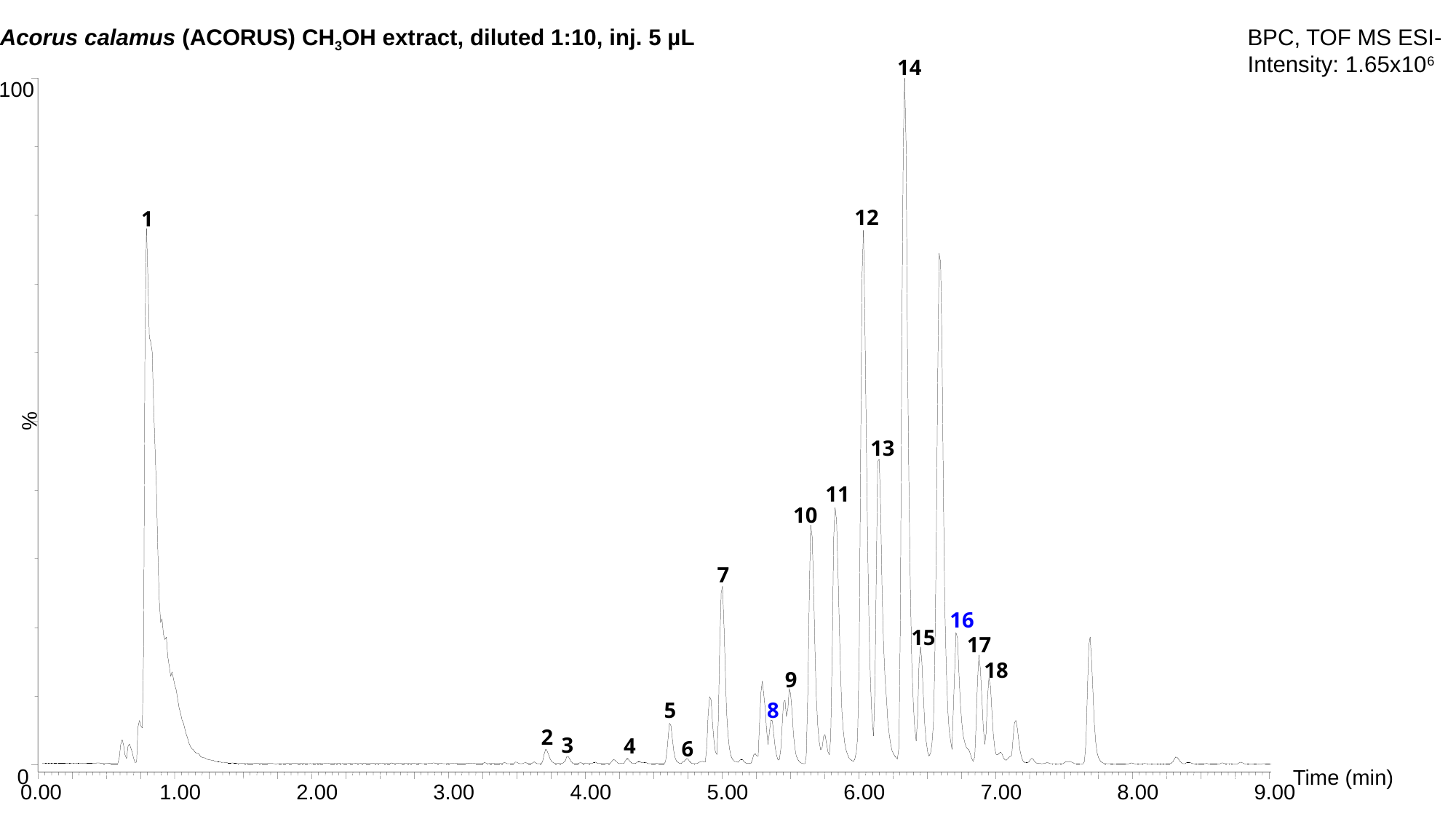

Acorus calamus (ACORUS) CH3OH extract, diluted 1:10, inj. 5 µL
BPC, TOF MS ESI-
Intensity: 1.65x10^6^
14
100
12
1
%
13
11
10
7
16
15
17
18
9
5
8
2
3
4
6
0
Time (min)
0.00
1.00
2.00
3.00
4.00
5.00
6.00
7.00
8.00
9.00

## Slide 4
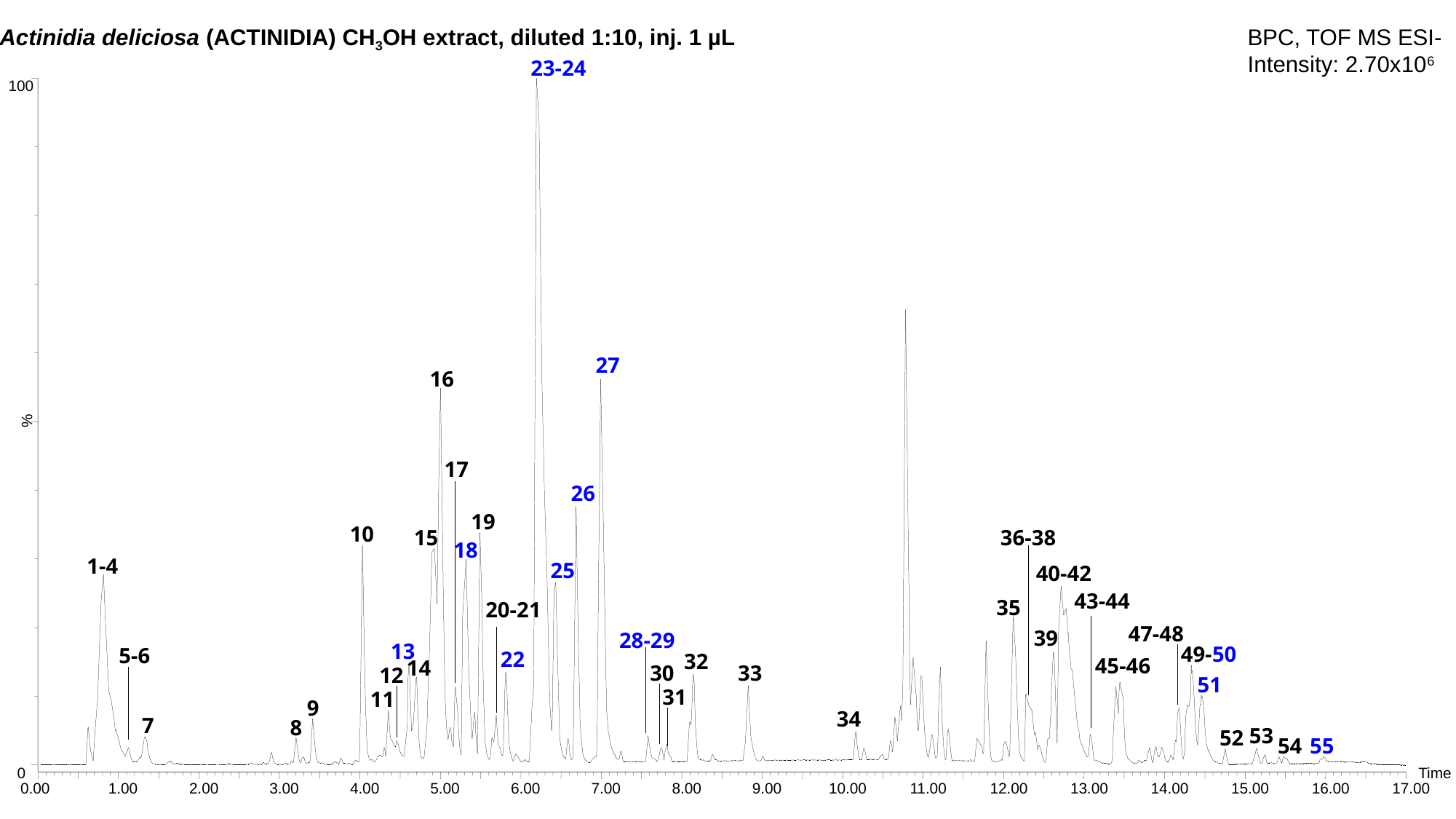

Actinidia deliciosa (ACTINIDIA) CH3OH extract, diluted 1:10, inj. 1 µL
BPC, TOF MS ESI-
Intensity: 2.70x10^6^
23-24
100
27
16
%
17
26
19
10
15
36-38
18
1-4
25
40-42
43-44
35
20-21
47-48
39
28-29
13
49-50
5-6
22
32
45-46
14
30
33
12
51
31
11
9
34
7
8
53
52
54
55
0
Time
0.00
1.00
2.00
3.00
4.00
5.00
6.00
7.00
8.00
9.00
10.00
11.00
12.00
13.00
14.00
15.00
16.00
17.00

## Slide 5
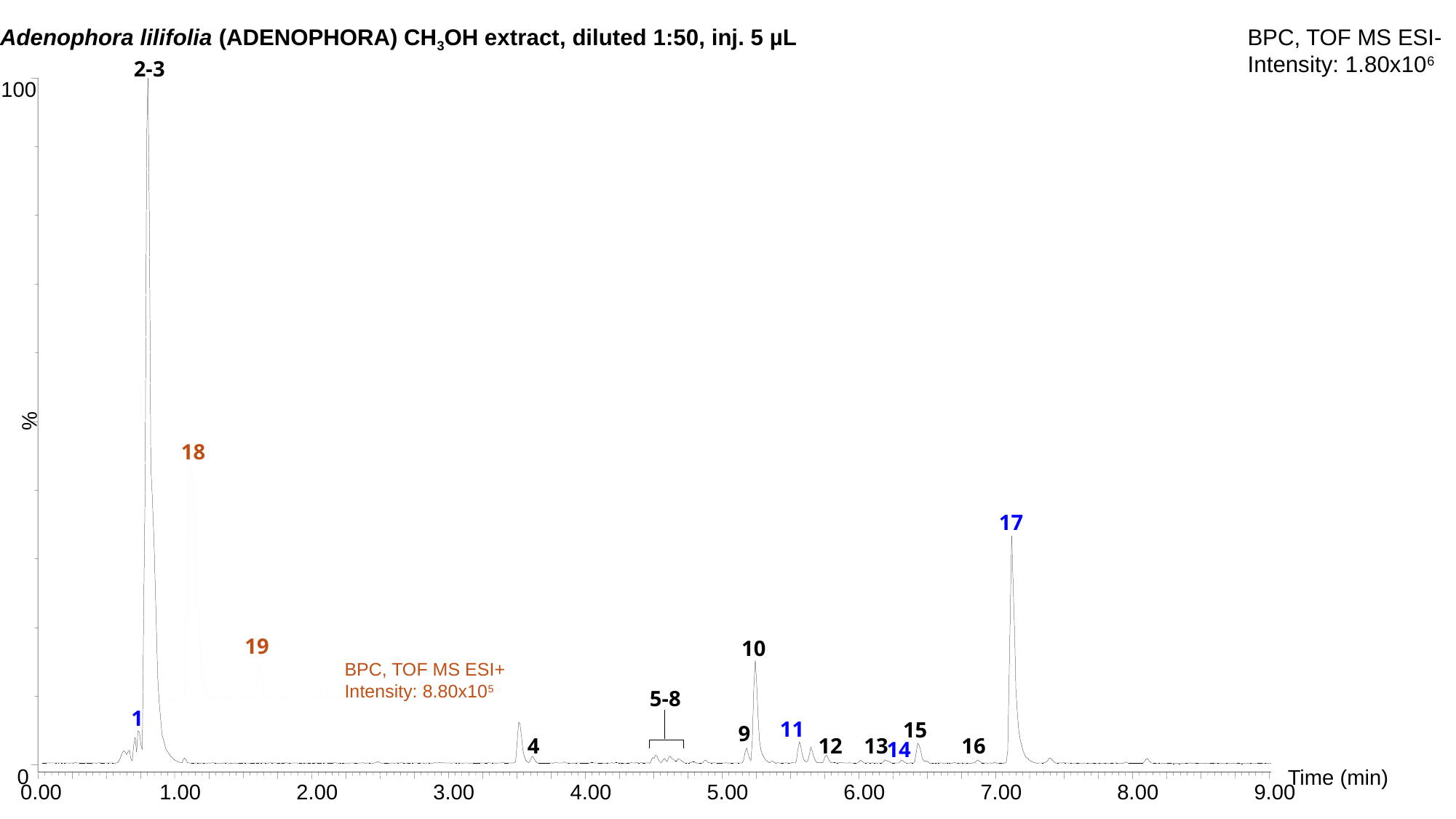

Adenophora lilifolia (ADENOPHORA) CH3OH extract, diluted 1:50, inj. 5 µL
BPC, TOF MS ESI-
Intensity: 1.80x10^6^
2-3
100
%
18
17
19
10
BPC, TOF MS ESI+
Intensity: 8.80x10^5^
5-8
1
11
15
9
4
12
13
16
14
0
Time (min)
0.00
1.00
2.00
3.00
4.00
5.00
6.00
7.00
8.00
9.00

## Slide 6
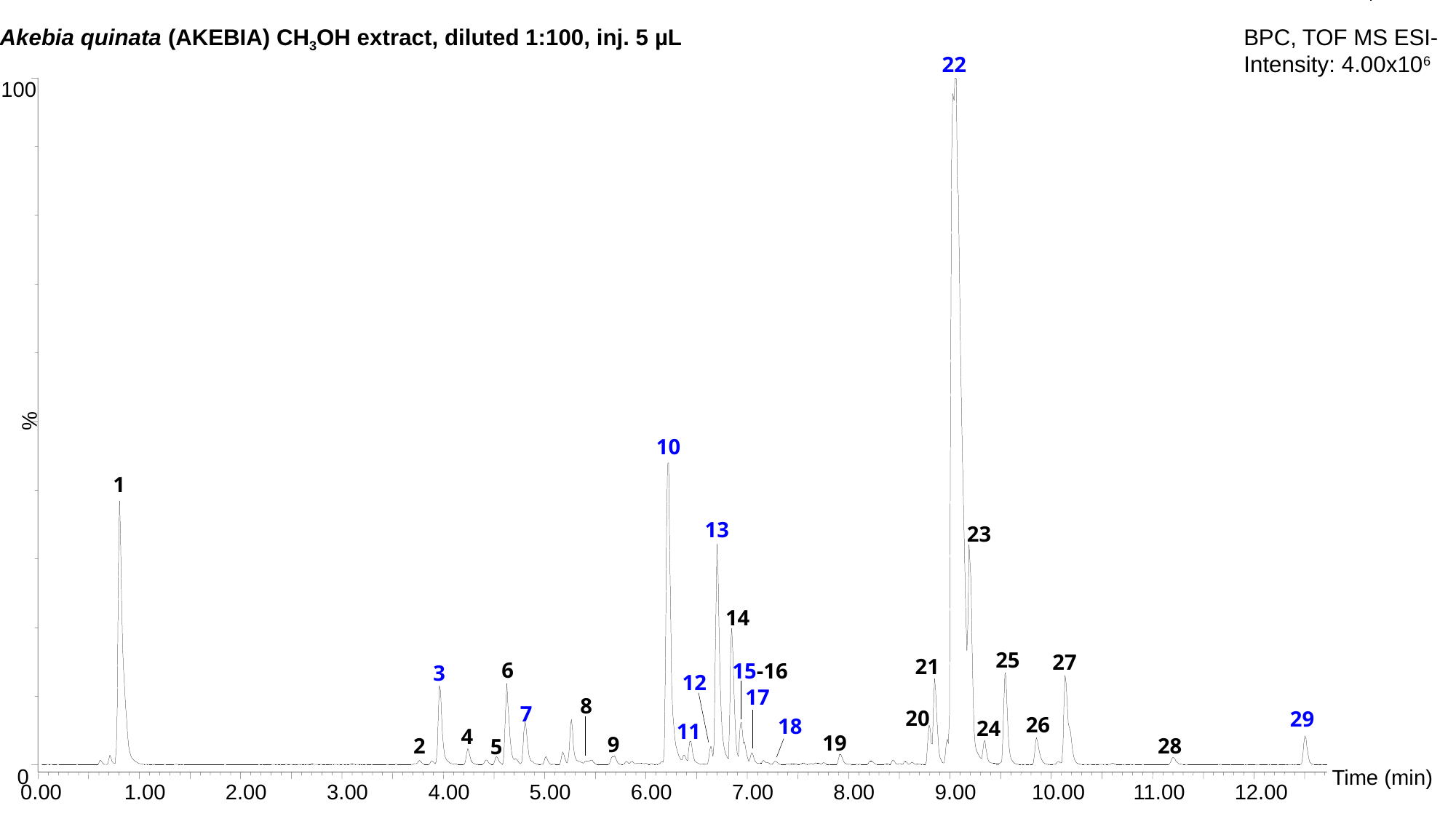

Akebia quinata (AKEBIA) CH3OH extract, diluted 1:100, inj. 5 µL
BPC, TOF MS ESI-
Intensity: 4.00x10^6^
22
100
%
10
1
13
23
14
25
27
21
6
15-16
3
12
17
8
7
20
29
26
18
24
11
4
19
9
2
28
5
0
Time (min)
0.00
1.00
2.00
3.00
4.00
5.00
6.00
7.00
8.00
9.00
10.00
11.00
12.00

## Slide 7
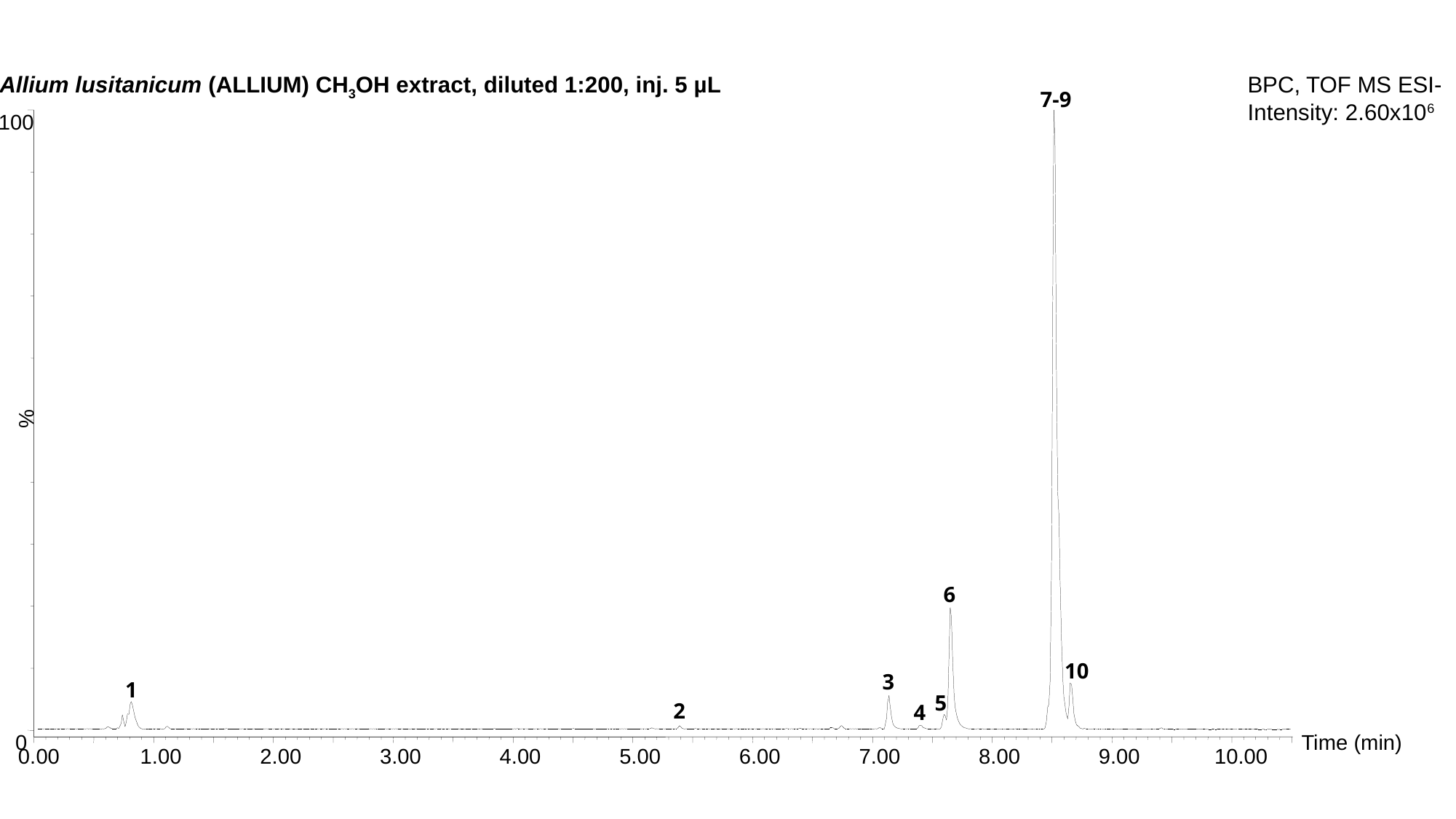

Allium lusitanicum (ALLIUM) CH3OH extract, diluted 1:200, inj. 5 µL
BPC, TOF MS ESI-
Intensity: 2.60x10^6^
7-9
100
%
6
10
3
1
5
2
4
0
Time (min)
0.00
1.00
2.00
3.00
4.00
5.00
6.00
7.00
8.00
9.00
10.00

## Slide 8
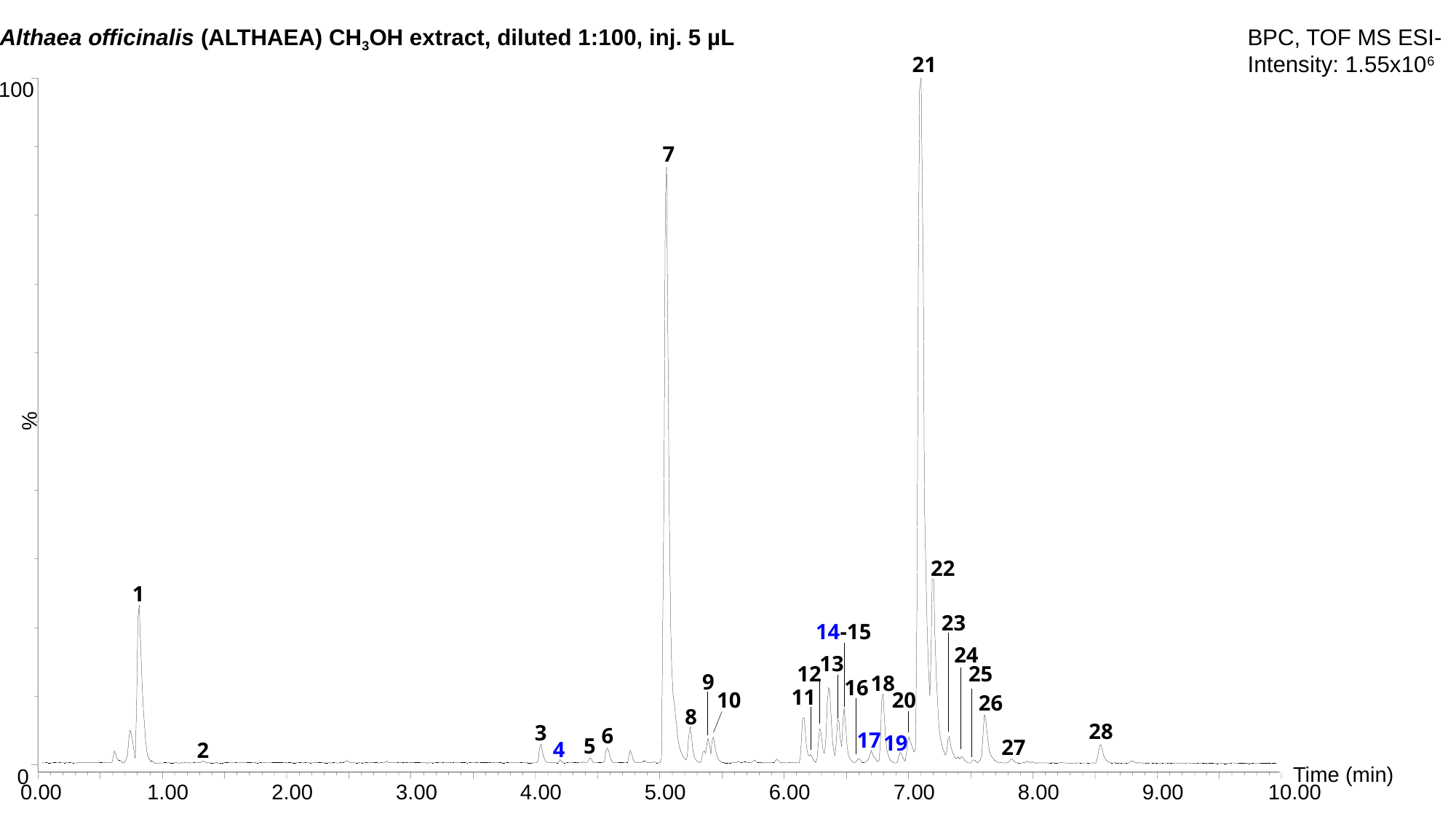

Althaea officinalis (ALTHAEA) CH3OH extract, diluted 1:100, inj. 5 µL
BPC, TOF MS ESI-
Intensity: 1.55x10^6^
21
100
7
%
22
1
23
14-15
24
13
25
12
9
18
16
11
10
20
26
8
28
3
6
17
19
5
27
4
2
Time (min)
0
0.00
1.00
2.00
3.00
4.00
5.00
6.00
7.00
8.00
9.00
10.00

## Slide 9
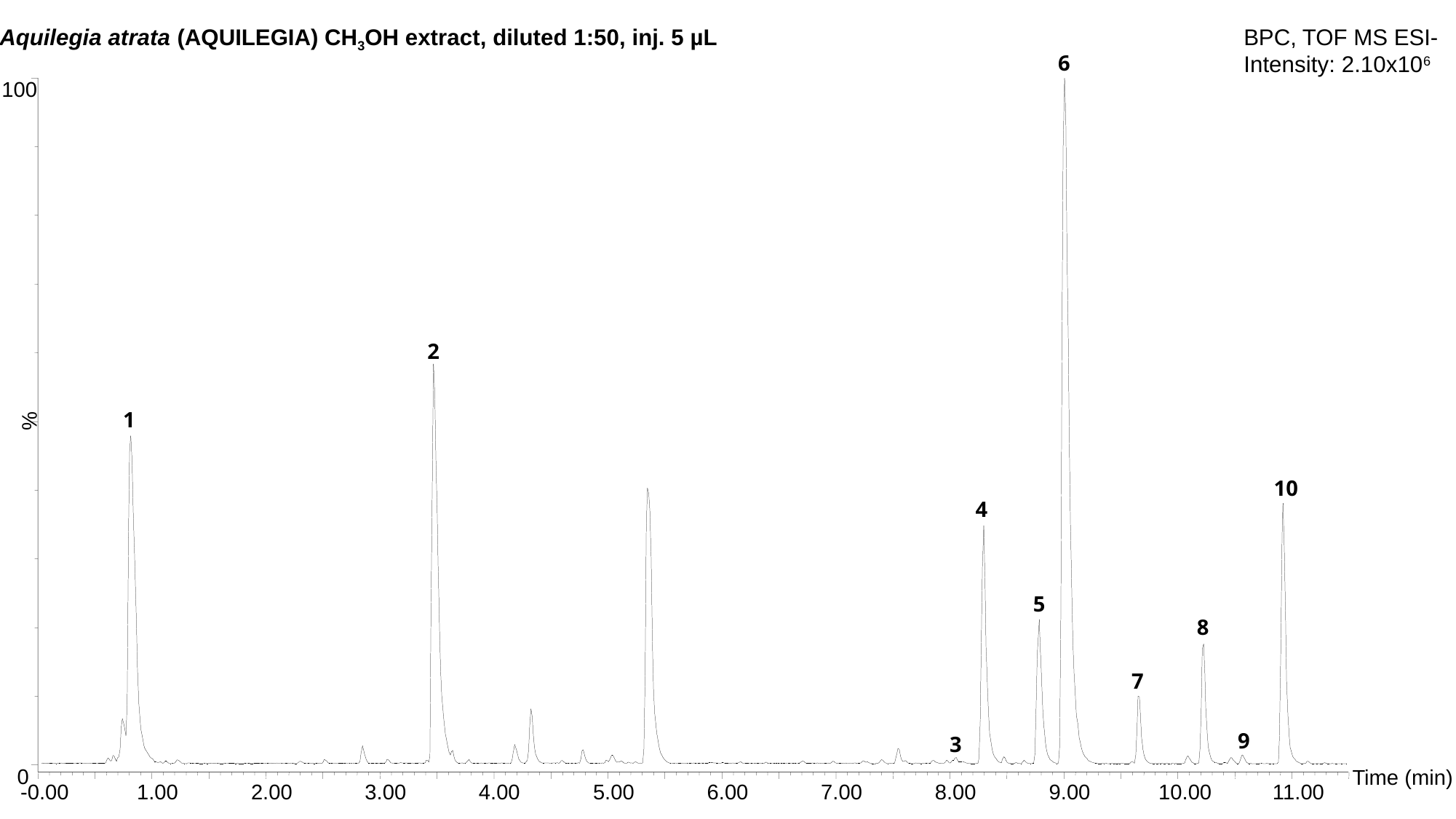

Aquilegia atrata (AQUILEGIA) CH3OH extract, diluted 1:50, inj. 5 µL
BPC, TOF MS ESI-
Intensity: 2.10x10^6^
6
100
2
1
%
10
4
5
8
7
9
3
0
Time (min)
-0.00
1.00
2.00
3.00
4.00
5.00
6.00
7.00
8.00
9.00
10.00
11.00

## Slide 10
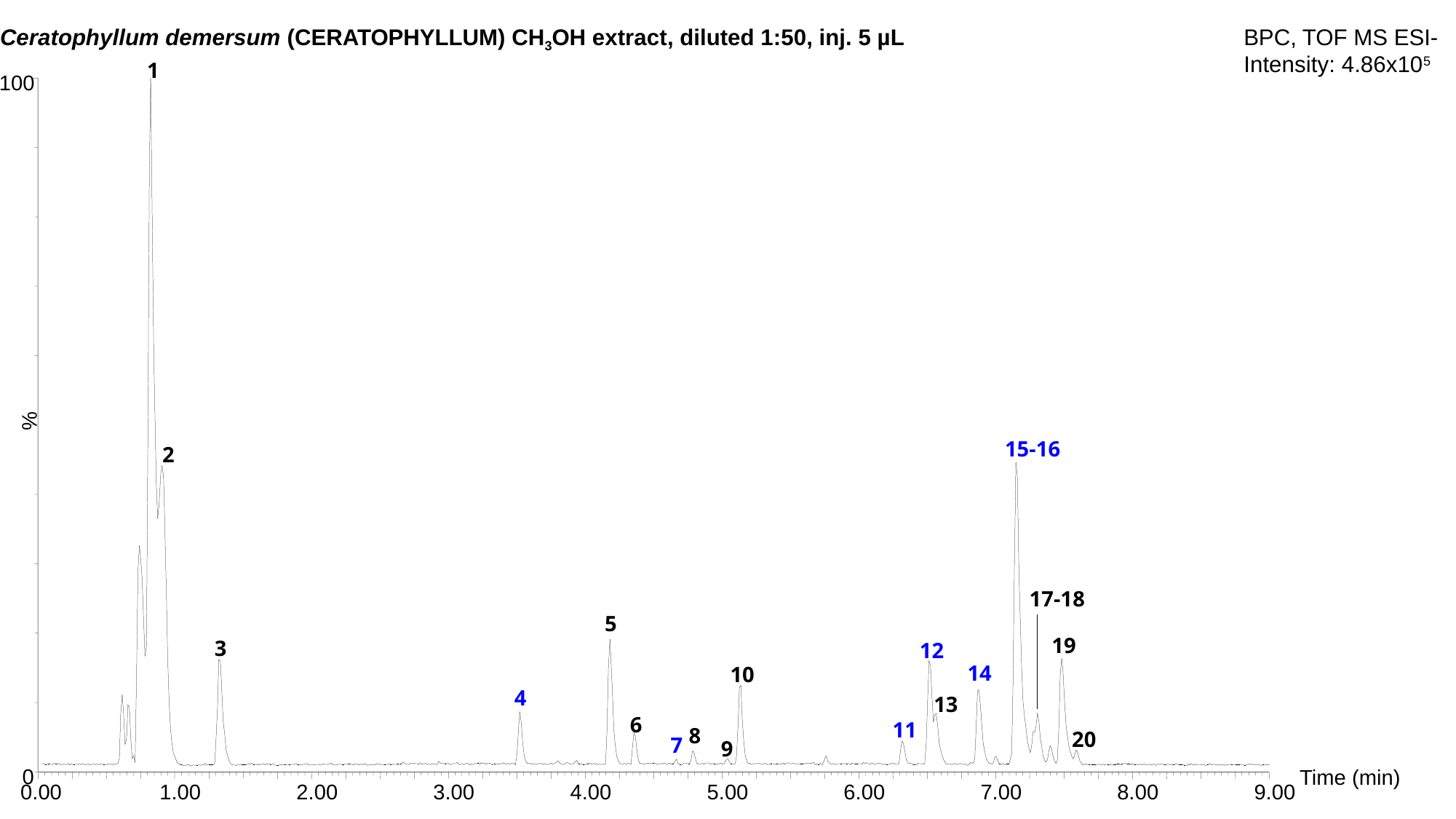

Ceratophyllum demersum (CERATOPHYLLUM) CH3OH extract, diluted 1:50, inj. 5 µL
BPC, TOF MS ESI-
Intensity: 4.86x10^5^
1
100
%
15-16
2
17-18
5
19
3
12
14
10
4
13
6
11
8
20
7
9
0
Time (min)
0.00
1.00
2.00
3.00
4.00
5.00
6.00
7.00
8.00
9.00

## Slide 11
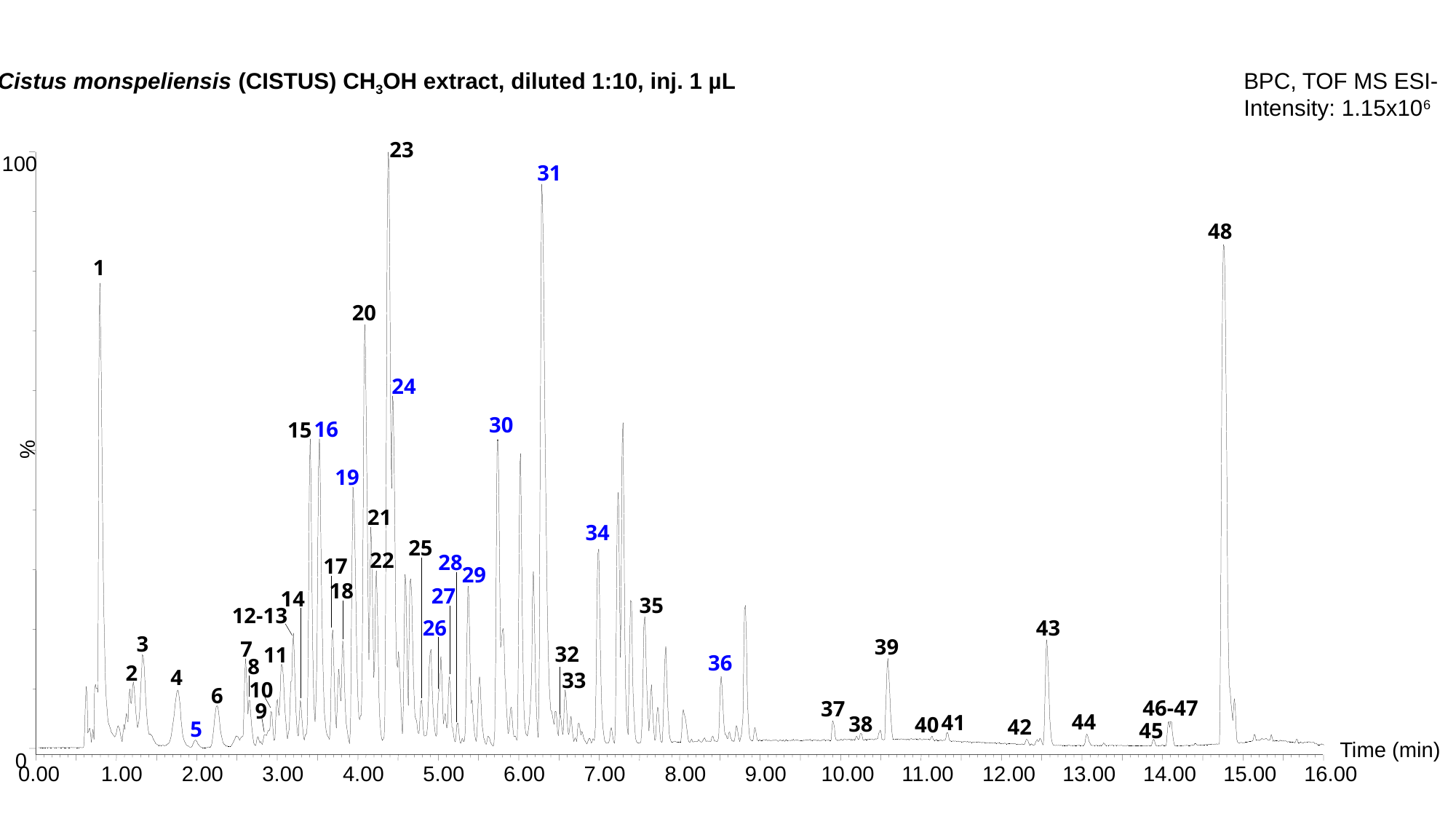

Cistus monspeliensis (CISTUS) CH3OH extract, diluted 1:10, inj. 1 µL
BPC, TOF MS ESI-
Intensity: 1.15x10^6^
23
100
31
48
1
20
24
30
16
15
%
19
21
34
25
22
28
17
29
18
27
14
35
12-13
26
43
3
39
7
32
11
36
8
2
4
33
10
6
46-47
37
9
44
41
38
40
42
5
45
Time (min)
0
0.00
1.00
2.00
3.00
4.00
5.00
6.00
7.00
8.00
9.00
10.00
11.00
12.00
13.00
14.00
15.00
16.00

## Slide 12
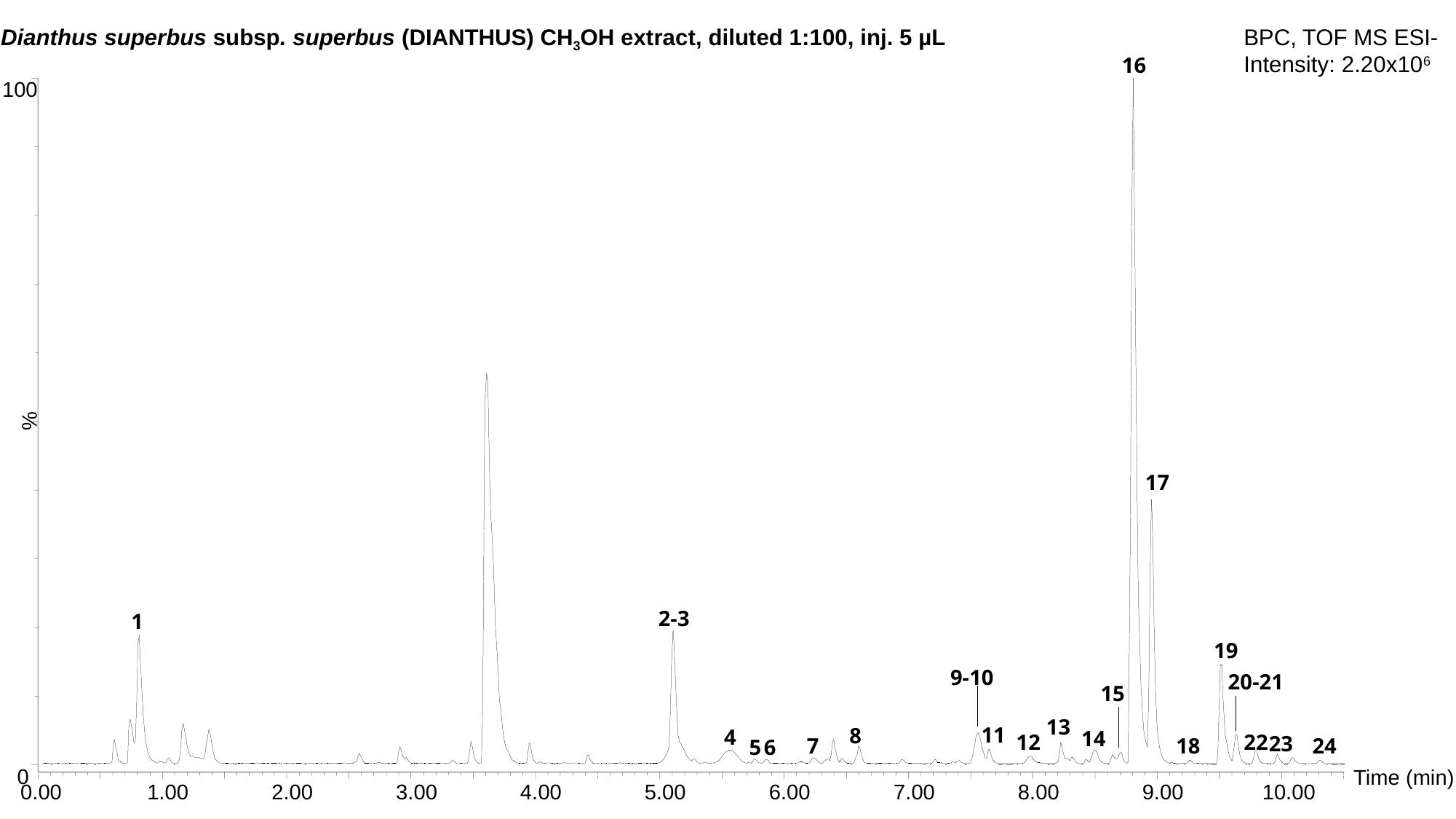

Dianthus superbus subsp. superbus (DIANTHUS) CH3OH extract, diluted 1:100, inj. 5 µL
BPC, TOF MS ESI-
Intensity: 2.20x10^6^
16
100
%
17
2-3
1
19
9-10
20-21
15
13
11
8
4
14
22
12
23
7
18
24
5
6
0
Time (min)
0.00
1.00
2.00
3.00
4.00
5.00
6.00
7.00
8.00
9.00
10.00

## Slide 13
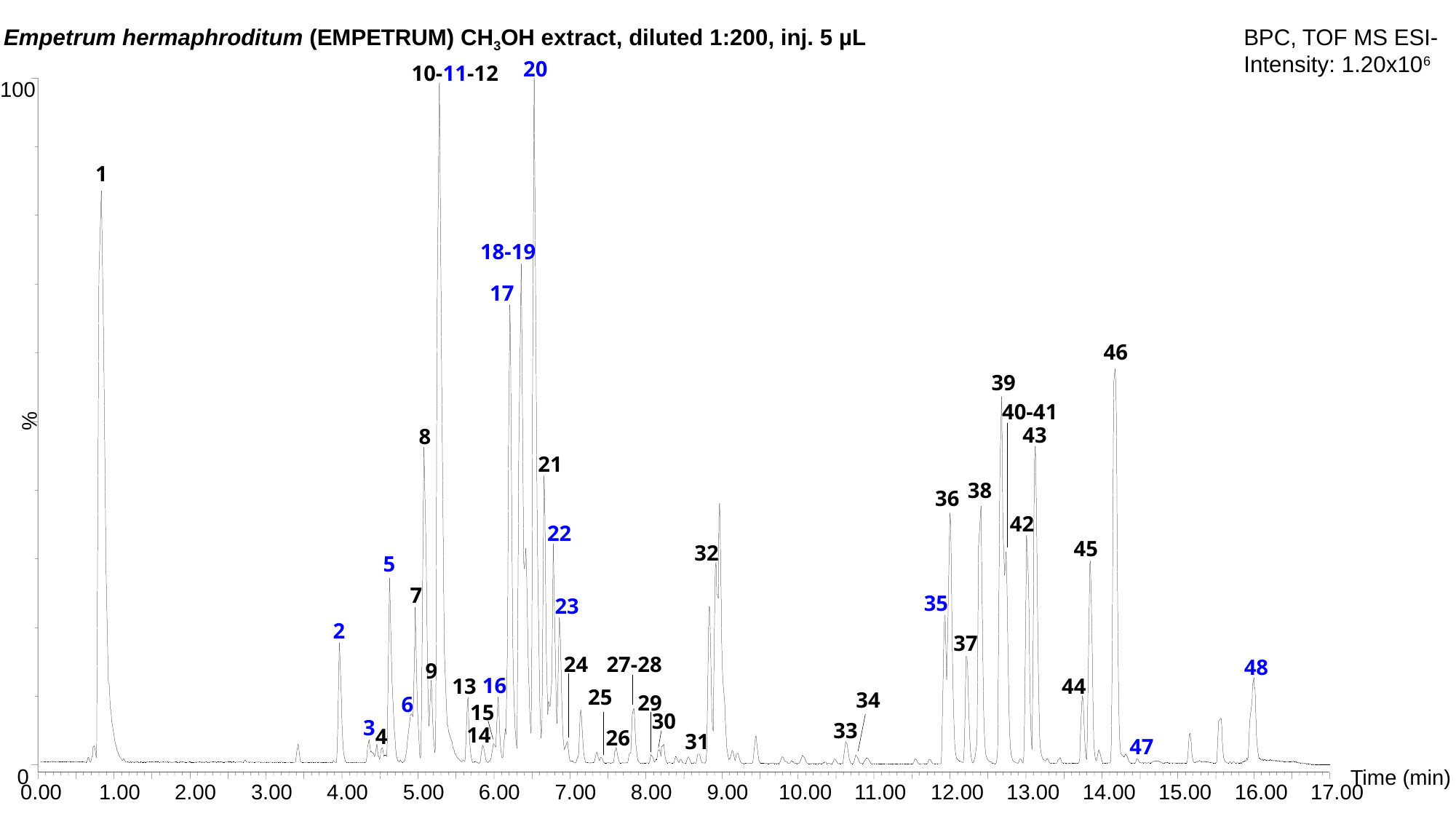

Empetrum hermaphroditum (EMPETRUM) CH3OH extract, diluted 1:200, inj. 5 µL
BPC, TOF MS ESI-
Intensity: 1.20x10^6^
20
10-11-12
100
1
18-19
17
46
39
40-41
%
43
8
21
38
36
42
22
45
32
5
7
35
23
2
37
24
27-28
48
9
16
13
44
25
34
29
6
15
30
3
33
14
4
26
31
47
0
Time (min)
0.00
1.00
2.00
3.00
4.00
5.00
6.00
7.00
8.00
9.00
10.00
11.00
12.00
13.00
14.00
15.00
16.00
17.00

## Slide 14
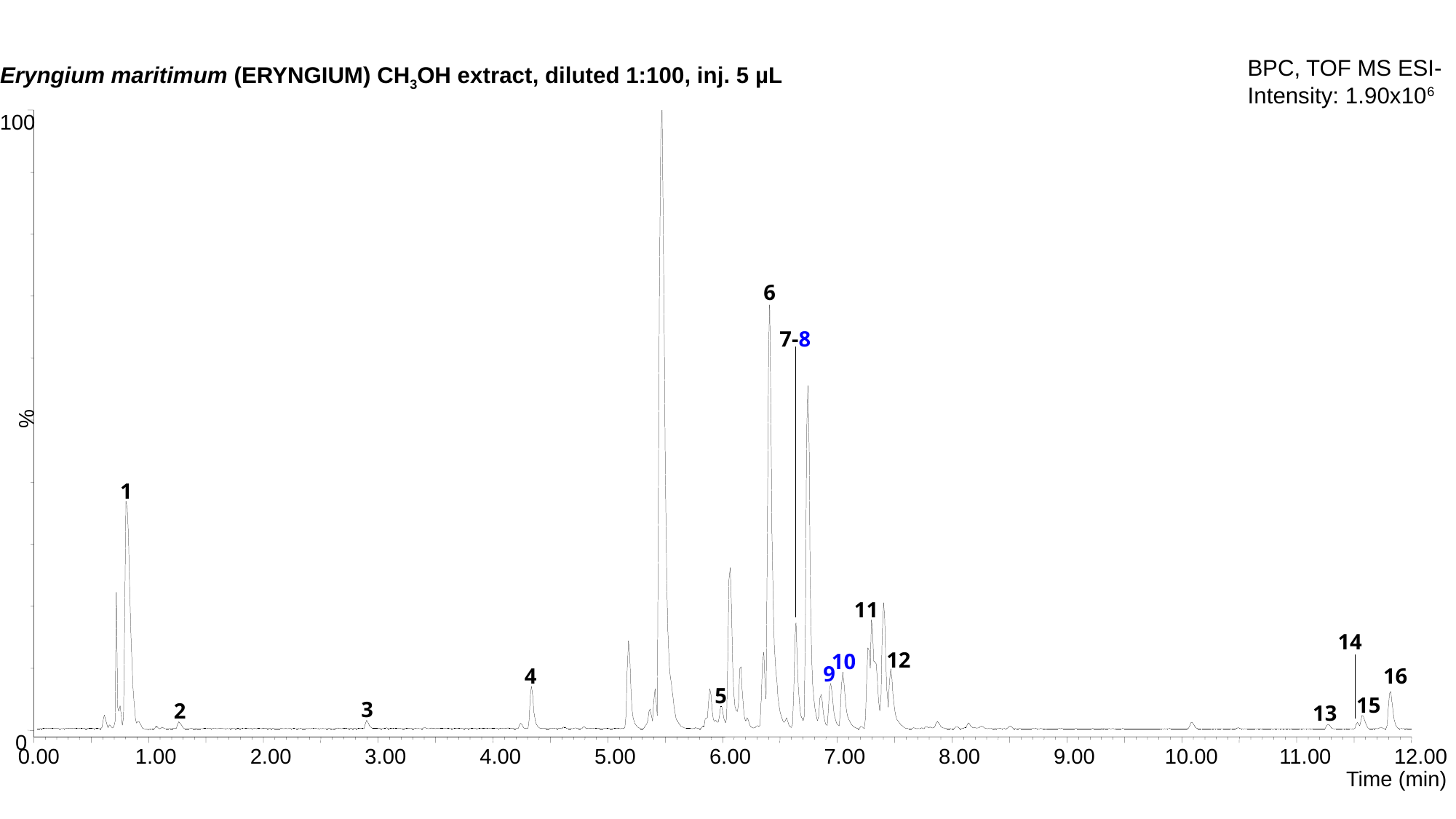

BPC, TOF MS ESI-
Intensity: 1.90x10^6^
Eryngium maritimum (ERYNGIUM) CH3OH extract, diluted 1:100, inj. 5 µL
100
%
0
0.00
1.00
2.00
3.00
4.00
5.00
6.00
7.00
8.00
9.00
10.00
11.00
12.00
Time (min)
6
7-8
1
11
14
12
10
9
4
16
5
15
3
2
13

## Slide 15
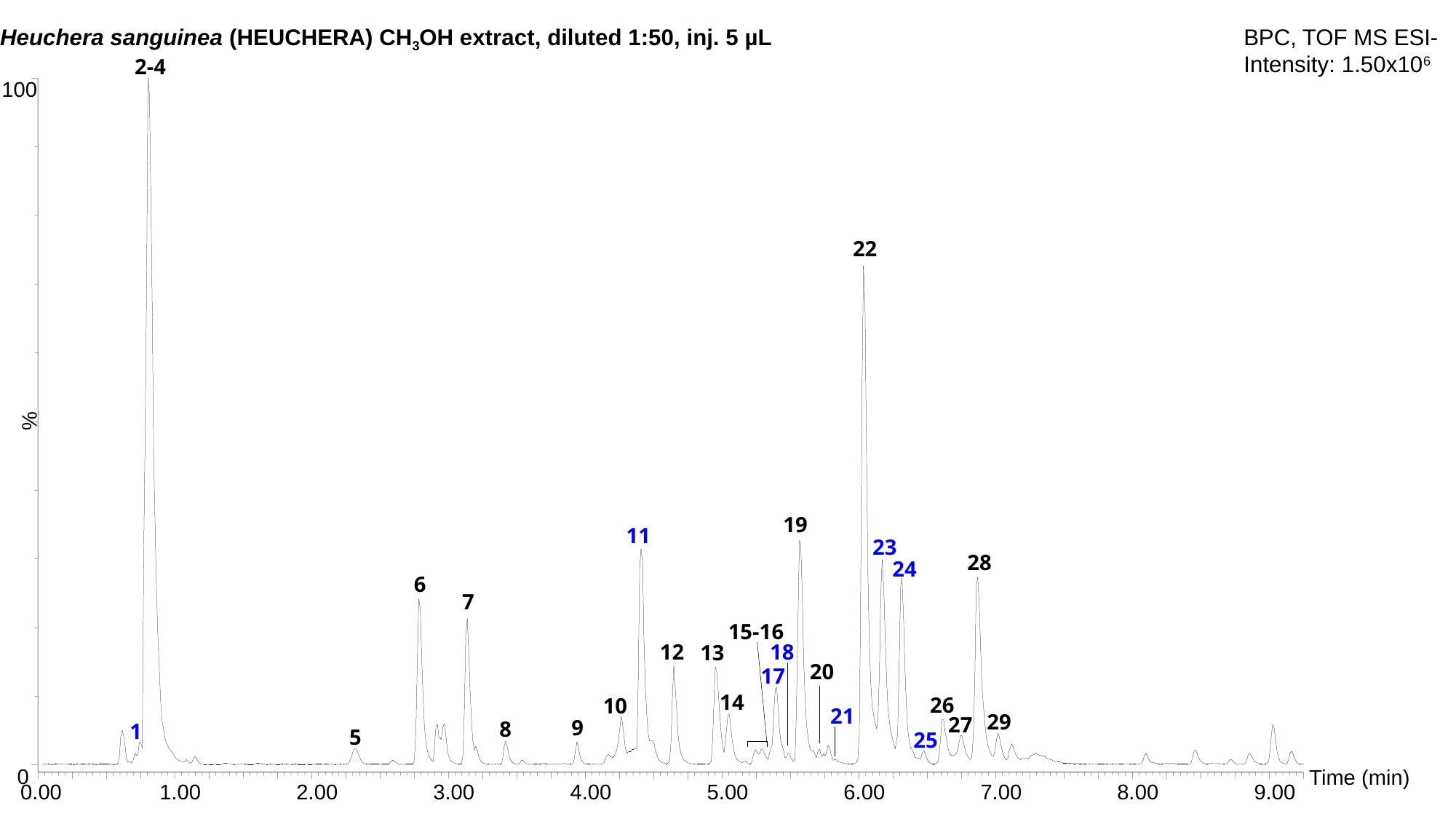

Heuchera sanguinea (HEUCHERA) CH3OH extract, diluted 1:50, inj. 5 µL
BPC, TOF MS ESI-
Intensity: 1.50x10^6^
2-4
100
22
%
19
11
23
28
24
6
7
15-16
12
18
13
20
17
14
26
10
21
29
27
9
8
1
5
25
0
Time (min)
0.00
1.00
2.00
3.00
4.00
5.00
6.00
7.00
8.00
9.00

## Slide 16
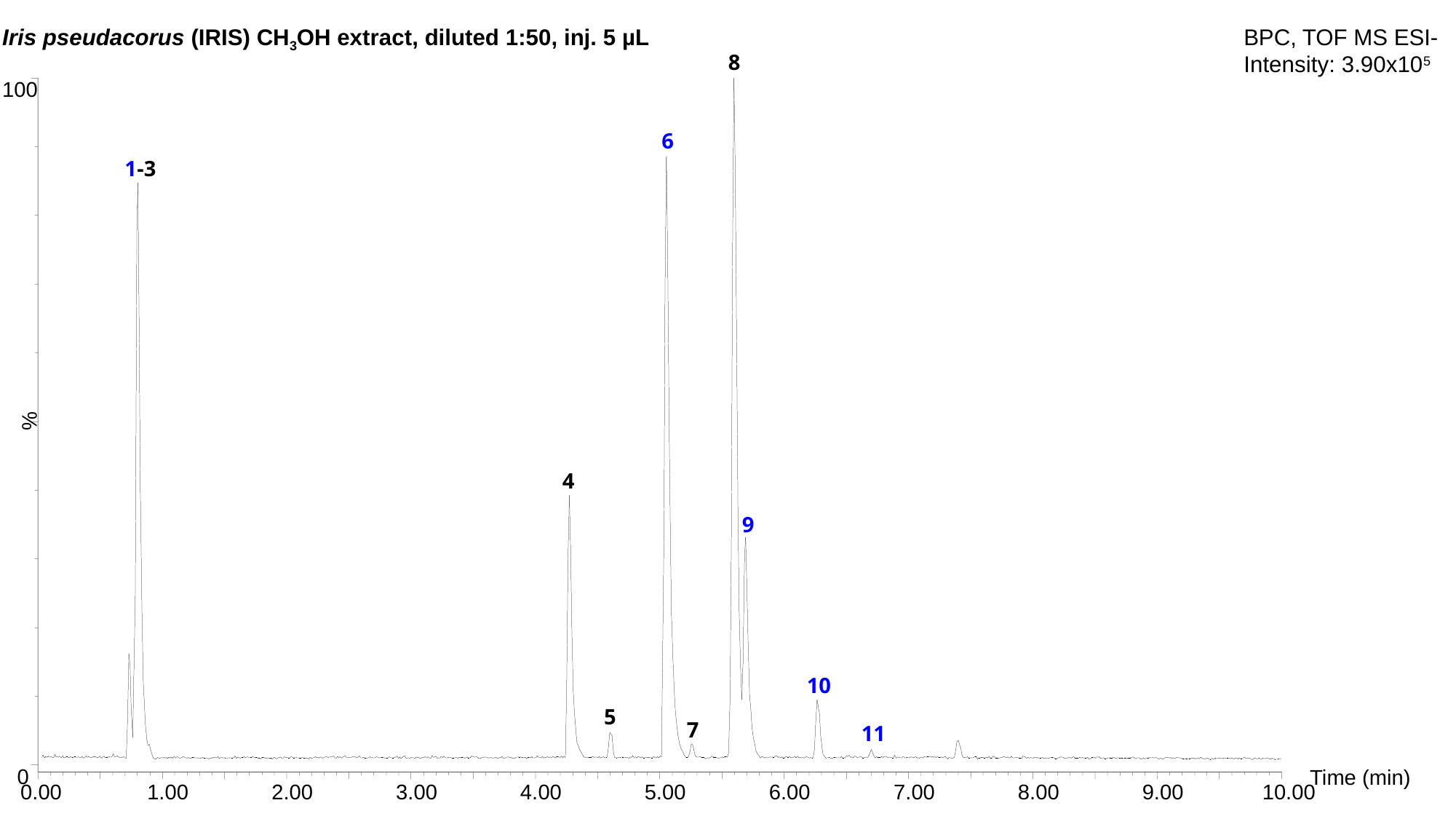

Iris pseudacorus (IRIS) CH3OH extract, diluted 1:50, inj. 5 µL
BPC, TOF MS ESI-
Intensity: 3.90x10^5^
8
100
6
1-3
%
4
9
10
5
7
11
0
Time (min)
0.00
1.00
2.00
3.00
4.00
5.00
6.00
7.00
8.00
9.00
10.00

## Slide 17
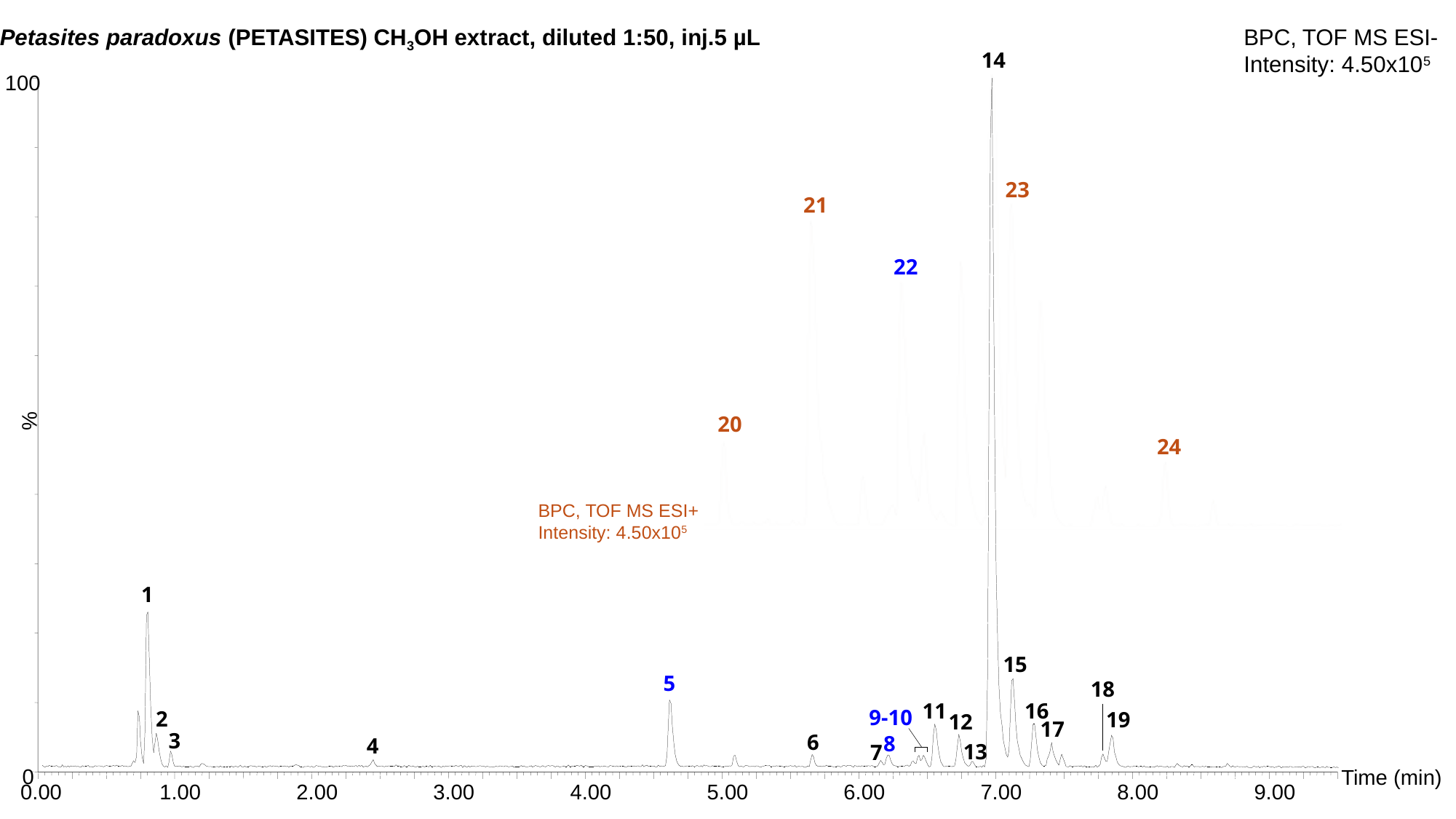

Petasites paradoxus (PETASITES) CH3OH extract, diluted 1:50, inj.5 µL
BPC, TOF MS ESI-
Intensity: 4.50x10^5^
14
100
23
21
22
20
%
24
BPC, TOF MS ESI+
Intensity: 4.50x10^5^
1
15
5
18
16
11
9-10
2
19
12
17
3
6
8
4
13
7
0
Time (min)
0.00
1.00
2.00
3.00
4.00
5.00
6.00
7.00
8.00
9.00

## Slide 18
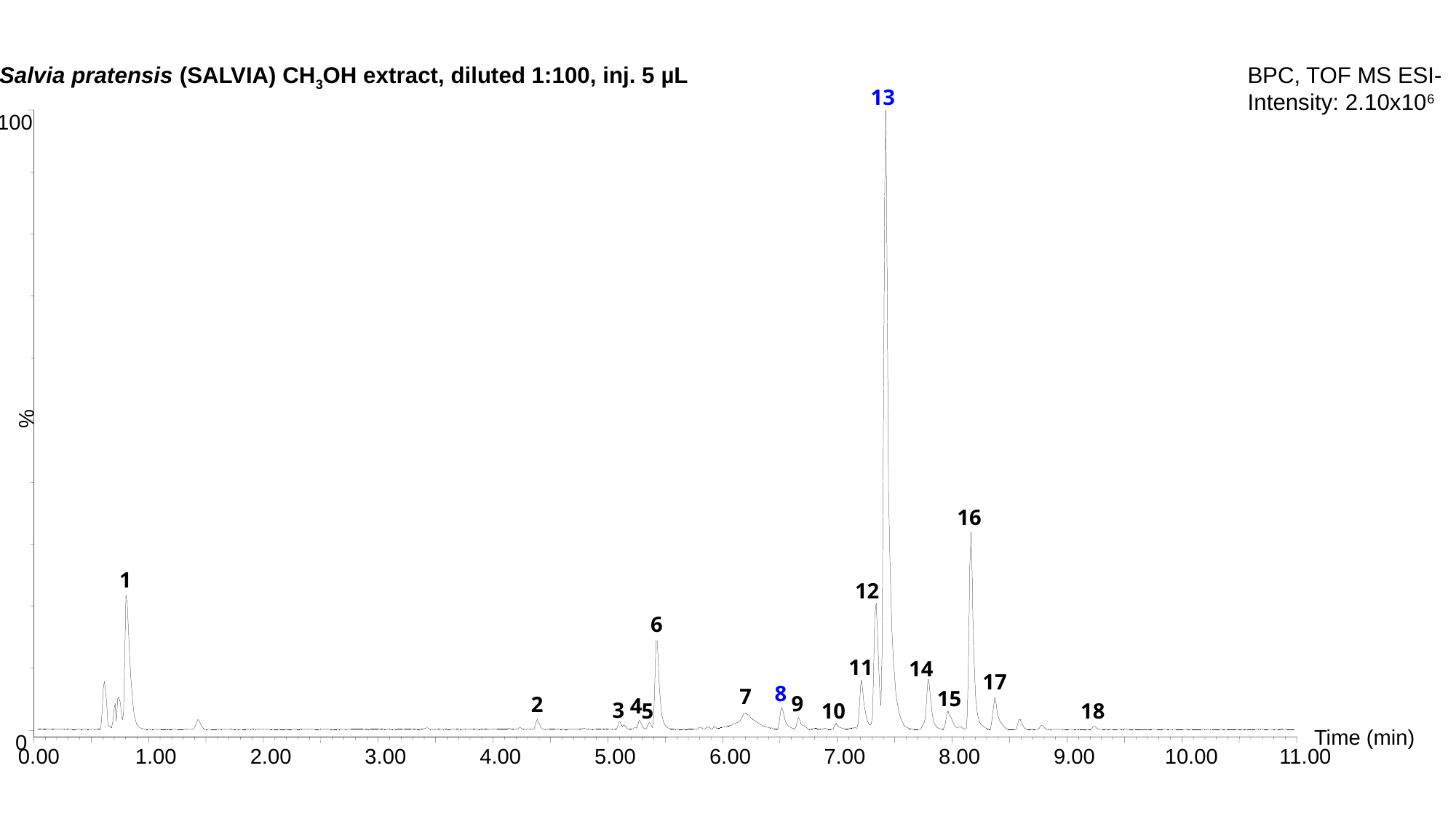

Salvia pratensis (SALVIA) CH3OH extract, diluted 1:100, inj. 5 µL
BPC, TOF MS ESI-
Intensity: 2.10x10^6^
13
100
%
16
1
12
6
11
14
17
8
7
15
9
2
4
3
5
10
18
Time (min)
0
0.00
1.00
2.00
3.00
4.00
5.00
6.00
7.00
8.00
9.00
10.00
11.00

## Slide 19
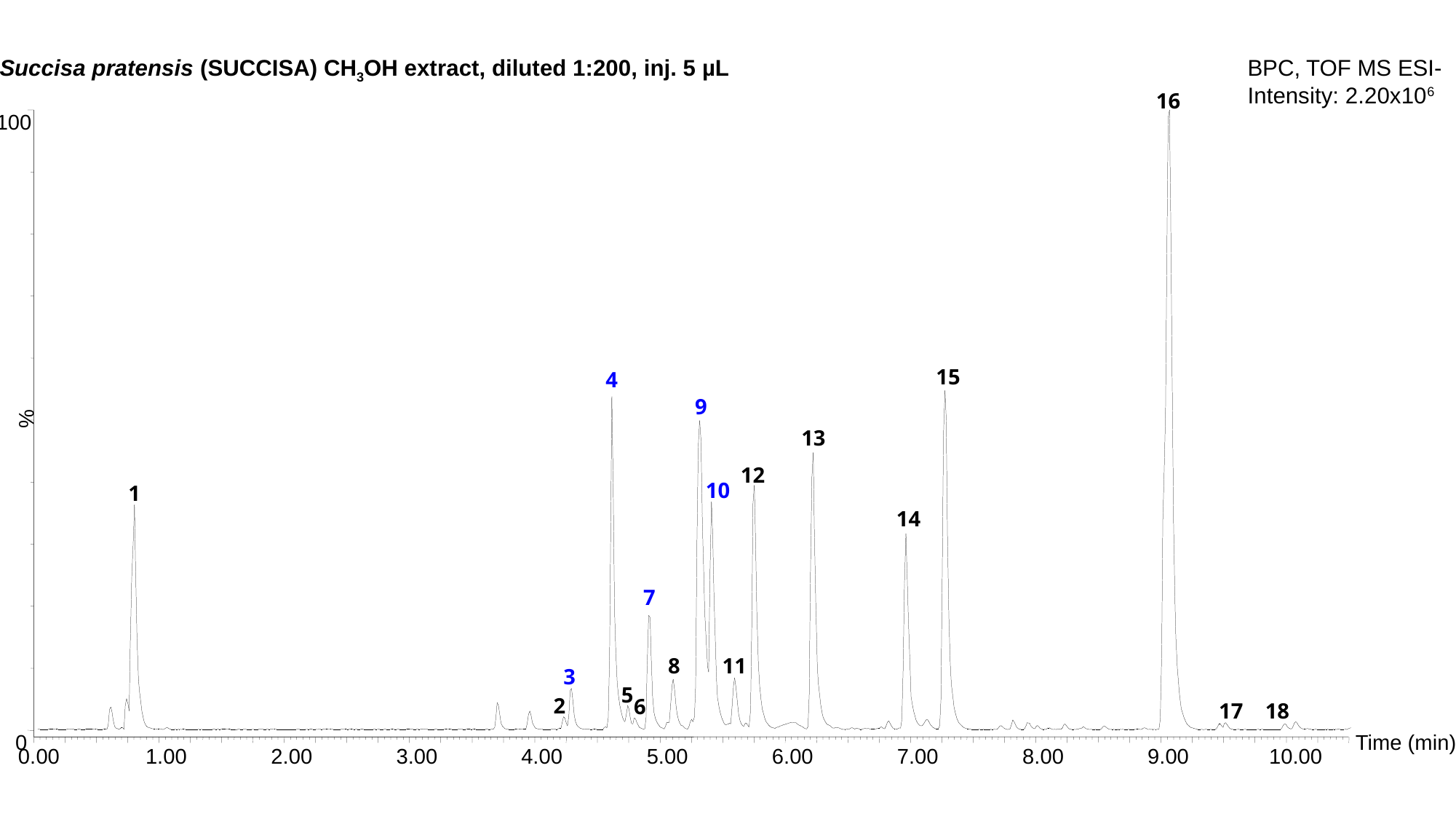

Succisa pratensis (SUCCISA) CH3OH extract, diluted 1:200, inj. 5 µL
BPC, TOF MS ESI-
Intensity: 2.20x10^6^
16
100
15
4
9
%
13
12
10
1
14
7
8
11
3
5
2
6
17
18
0
Time (min)
0.00
1.00
2.00
3.00
4.00
5.00
6.00
7.00
8.00
9.00
10.00

## Slide 20
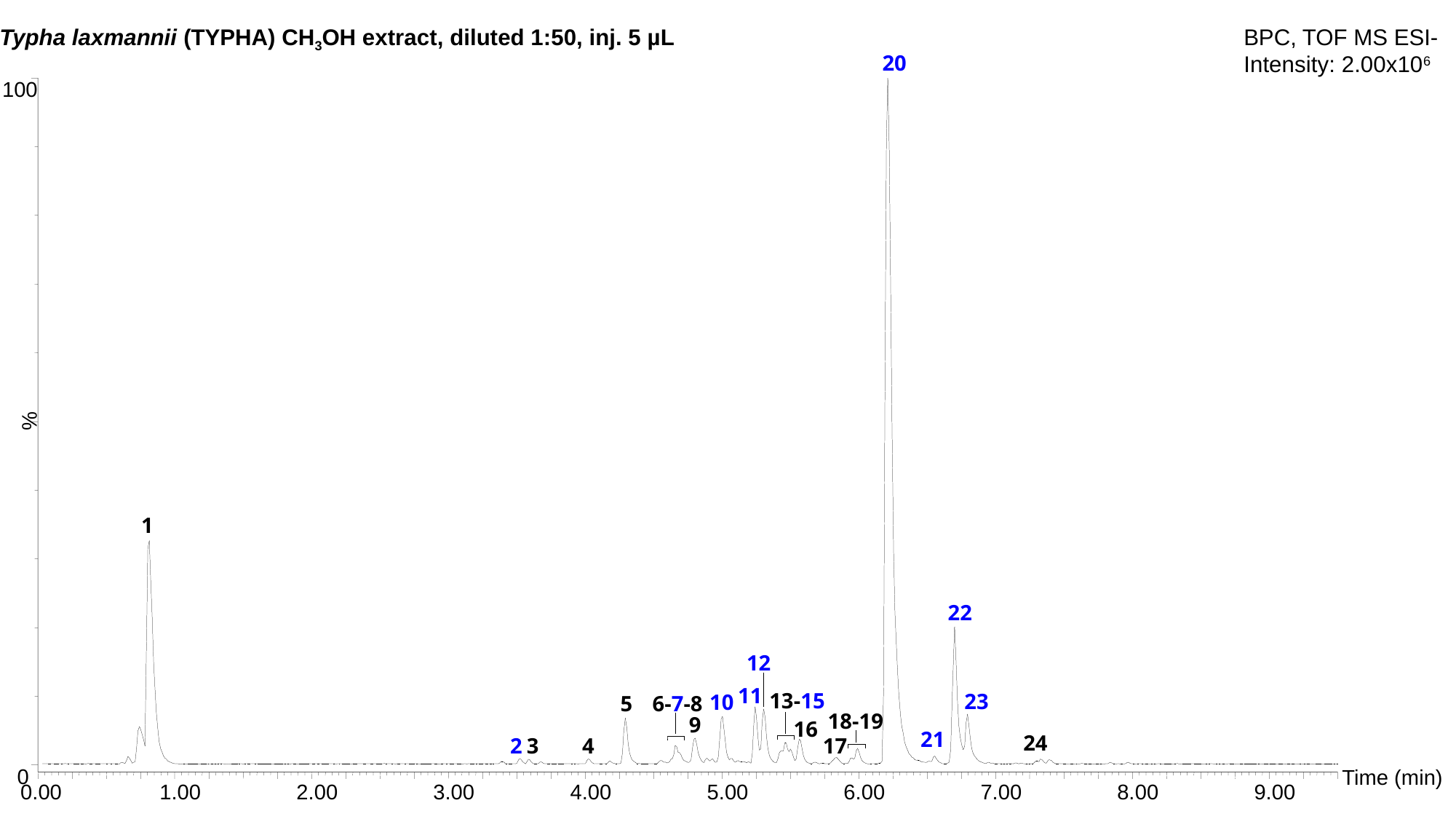

Typha laxmannii (TYPHA) CH3OH extract, diluted 1:50, inj. 5 µL
BPC, TOF MS ESI-
Intensity: 2.00x10^6^
20
100
%
1
22
12
11
13-15
23
10
6-7-8
5
18-19
9
16
21
2
3
4
17
0
Time (min)
0.00
1.00
2.00
3.00
4.00
5.00
6.00
7.00
8.00
9.00
24
