# Supplementary material for: Screening of Mediterranean Plant-Derived Extracts for Antioxidant Effect in Cell-Free and Human Cell Line Models
Source: Antioxidants (Basel). 2025 Oct 9;14(10):1217. doi: 10.3390/antiox14101217 (PMC12561960; doi:10.3390/antiox14101217)
Supplement: Supplementary file 1 [file antioxidants-14-01217-s001.zip › Supplementary Table S1 and Figures S1 and S2.pdf]

Supplementary Materials

Screening of Mediterranean Plant-Derived Extracts for Antioxidant Effect in Cell-Free and Human Cell Lines Models

Giuseppe Argentino [1,2,*,†], Edoardo Giuseppe Di Leo [1,2,†], Chiara Stranieri [1], Stefano Negri [2,3], Mauro Commisso [2,3], Flavia Guzzo [2,3], Anna Maria Fratta Pasini [1,2], Annalisa Castagna [1] and Simonetta Friso [1,2]

[1] Department of Medicine, University of Verona, 37134 Verona, Italy; edoardogiuseppe.dileo@univr.it; chiara.stranieri@univr.it; annamaria.frattapasini@univr.it; annalisa.castagna@univr.it; simonetta.friso@univr.it;
[2] National Biodiversity Future Center (NBFC), 90133 Palermo, Italy;
[3] Department of Biotechnology, University of Verona, 37134 Verona, Italy; stefano.negri@univr.it; mauro.commisso@univr.it; flavia.guzzo@univr.it;
[*] Correspondence: giuseppe.argentino@univr.it;
[†] These authors contributed equally to this work and share first authorship.

Supplementary Table and Figures

Table S1. Antioxidant activity of plant extract in THP-1 cells at concentration showing statistically significant effects.

Extract	Concentration (μg/mL)	ROS Reduction (% ± SD)	*p*-Value
ACALYPHA	5	65.18 ± 6.42	0.032
	25	67.47 ± 5.79	0.0427
ACTINIDIA	15	46.5 ± 27.07	0.0107
	25	48.69 ± 27.42	0.0306
ADENOPHORA	0.5	49.47 ± 31.73	0.0019
	5	58.56 ± 26.65	0.0111
	15	26.88 ± 16.84	<0.0001
	25	63.21 ± 31.04	0.0375
AKEBIA	15	51.38 ± 14.5	0.0406
	25	51.66 ± 11.99	0.0081
ALLIUM	5	61.43 ± 23.96	0.0038
	15	59.97 ± 15.07	0.0007
	25	67.5 ± 13.75	0.008
ALTHEA	0.5	49.11 ± 12.25	0.0029
	5	47.71 ± 21.13	0.001
	15	33.55 ± 13.47	<0.0001
	25	39.4 ± 11.58	<0.0001
AQUILEGIA	0.5	38.87 ± 18.36	0.0001
	5	35.96 ± 20.73	<0.0001
	15	47.72 ± 27.09	0.0011
	25	44.41 ± 26.36	0.0003
DIANTHUS	0.5	47.28 ± 17.66	<0.0001
	5	59.26 ± 28.81	0.0028
	15	35.1 ± 21.93	<0.0001
	25	47.31 ± 14.24	<0.0001
EMPETRUM	15	68.69 ± 22.7	0.0095

ERYNGIUM	15	39.82 ± 32.26	0.0013
	25	40.08 ± 29.11	0.0002
IRIS	0.5	54.12 ± 6.77	0.0275
	5	50.42 ± 12.5	0.0181
	15	54.27 ± 14.82	0.0337
PETASITES	0.5	52.05 ± 15.17	0.0013
	5	45.53 ± 8.25	<0.0001
	15	44.12 ± 13.42	<0.0001
	25	54.45 ± 19.81	0.0009
SALVIA	5	74.11 ± 15.86	0.0088
SUCCISA	0.5	57.75 ± 29.68	0.0006
	5	54.69 ± 28.26	0.0002
	15	61.48 ± 33.73	0.0023
	25	63.06 ± 39.57	0.0118
TYPHA	0.5	48.79 ± 26.57	0.0005
	5	54.77 ± 23.89	0.0063
	15	44.86 ± 26.78	<0.0001
	25	38.43 ± 16.78	<0.0001

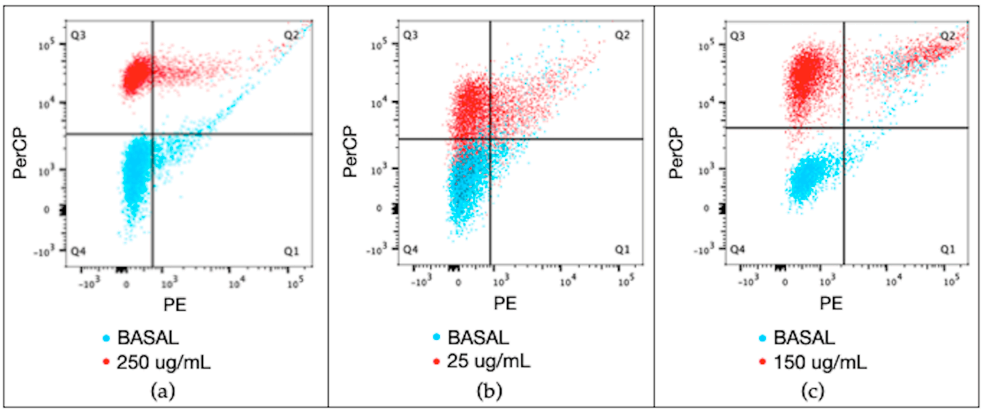

Figure S1. Example of the effect of a plant extract on THP-1 cells (**a**), HUVECs (**b**) and HIECs (**c**). The distribution of cells across the four quadrants indicates cytotoxicity at the indicated concetrations. (Q1 early apoptotic cells; Q2 late apoptotic cells; Q3 necrotic cells; Q4 viable cells) (7-AAD was detected in the PerCP channel, and AnnV in the PE channel).

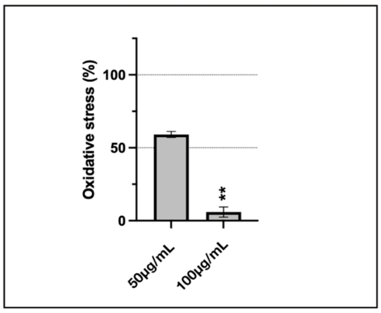

Figure S2. Antioxidant potential of plant extracts in HIECs. Significant antioxidant effects, indicated by a reduction in intracellular ROS levels compared to the positive control, were observed after incubation with ERYNGIUM at 100 μg/mL (** $p < 0.01$).
